# Repurposing radiosensitising medicines for radiotherapy: an overview

**DOI:** 10.1136/bmjonc-2023-000192

**Published:** 2024-01-17

**Authors:** Jie Man Low, Gonzalo Rodriguez-Berriguete, Geoff S Higgins

**Affiliations:** 1 Department of Oncology, Oxford University Hospitals NHS Trust, Oxford, UK; 2 Department of Oncology, University of Oxford, Oxford, UK

**Keywords:** Radiotherapy

## Abstract

Repurposing established non-cancer drugs for the treatment of cancer offers potential benefits such as speed of clinical translation and financial efficiencies. In this study, we assess the landscape of repurposing drugs for combined use with radiotherapy (RT) based on their capacity to increase tumour radiosensitivity. Using a literature-based approach, we identified 42 radiosensitising drugs with varied non-cancer indications and mechanisms of action, that have entered or completed clinical trials in combination with RT or with chemoradiotherapy. Two compounds, nicotinamide and nimorazole, have entered routine but limited clinical use in combination with radiotherapy. We provide an overview on these successfully repurposed drugs, and highlight some examples of unsuccessful repurposing efforts and drug candidates with an uncertain prospect of success. Upon reviewing the trials, we identified some common themes behind the unsuccessful efforts, including poor trial reporting, absence of biomarkers and patient selection, sub-optimal pharmacological properties, inappropriate trial design, lack or inadequate consideration of pre-clinical and clinical data, and limited funding support. We point out future directions to mitigate these issues and increase the likelihood of success in repurposing drug treatments for radiotherapy.

## Introduction

Radiotherapy (RT) is used in over half of patients with cancer, with the majority treated with curative intent.^1^ With the rapid advances in RT, coupled with the rising incidence of cancer, the number of patients undergoing RT is predicted to increase significantly.^2^ The development of radiosensitisers would hence benefit this expanding group of patients.

**Figure 1:**
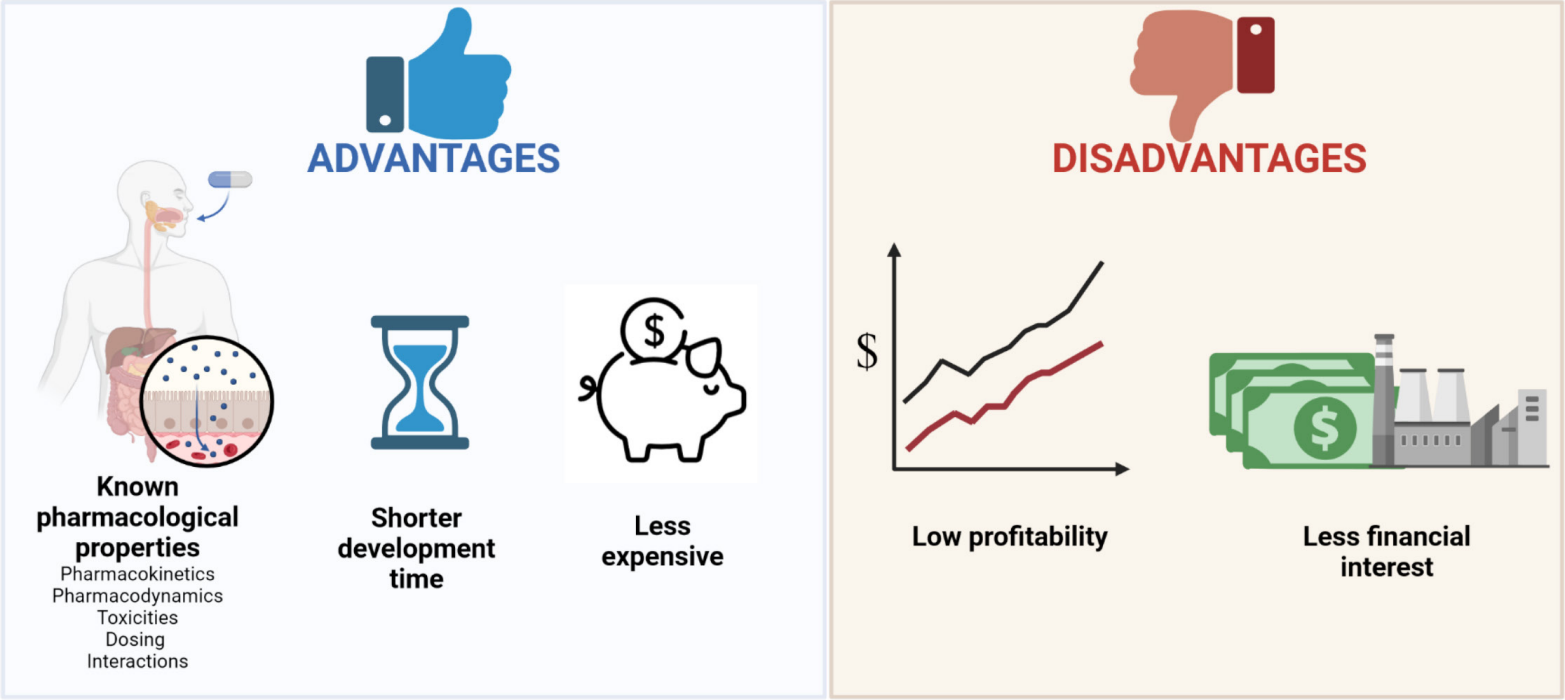
Advantages and disadvantages of repurposing drugs when compared with conventional drug development.

Drug repurposing is the process of discovering new uses for approved drugs that differ from the original medical indication. When a licence is granted to existing cancer drugs for new cancer indications, this is called ‘soft repurposing’. ‘Hard repurposing’ occurs when non-cancer drugs are used in cancer therapy.^3^ The theoretical advantages of repurposing over conventional drug development are plenty (figure 1). One of the appeals of repurposing lies in the wealth of knowledge on the pharmacological profile of existing drugs, which could derisk, accelerate and lower the costs of clinical testing. While de novo drug development is estimated to take 13–17 years, repurposing a drug would only require 3–12 years and cost up to 85% less.^4 5^ Furthermore, since many of these drugs are generic, the cost to the health providers or final users is expected to be much cheaper than that of patented drugs.^6^ This review aims to explore the ‘hard repurposing’ of drugs used to enhance tumour radiosensitivity—and thus, improve RT efficacy—by outlining past and ongoing clinical trials, followed by a discussion on the challenges and future directions in the repurposing of these radiosensitising drugs.

## Search criteria

We adopted a search methodology similar to a previous systematic review on trials testing repurposed drugs.^7^ We divided the search into two stages to identify drugs that fulfil the following: have a non-cancer indication, proposed to have radiosensitising activity and have been investigated in clinical trials. First, we gathered a list of non-cancer drugs with anticancer properties, mainly from the Repurposing Drugs in Oncology (ReDO) database (https://www.anticancerfund.org/en/redo-db; website last updated 3 August 2023; assessed between 20 May 2023 and 15 August 2023).^8^ Additionally, we used the bibliographic databases PubMed (https://pubmed.ncbi.nlm.nih.gov/) and the NHS Knowledge and Library Hub (https://library.nhs.uk/knowledgehub/) between 20 May 2023 and 10 June 2023 to identify other drugs not found on the ReDO database, using the terms ‘repurposing’, ‘cancer’ and ‘radiotherapy’. We also included drugs not discovered through this strategy but known to us to meet the above criteria. We have excluded treatment modalities that are not medications, such as hyperbaric oxygen, and drugs investigated with the expectation of alleviating side effects of RT rather than increasing radiosensitivity. Observational studies were not included.

Second, we identified which of these drugs are being or have been investigated in clinical trials in combination with RT and compiled the information on past and ongoing clinical trials for each. We searched on PubMed and on three trial registers—ClinicalTrials.gov (https://clinicaltrials.gov/), WHO International Clinical Trials Registry Platform (WHO ICTRP) (https://trialsearch.who.int/) and the International Standard Randomised Controlled Trial Number (ISRCTN) (https://www.isrctn.com/search?q=). In ClinicalTrials.gov, we included the search term “cancer” under the “Condition or disease” tab, and “[drug name] AND (radiation therapy OR radiotherapy)” under the “Other terms” tab. In WHO ICTRP, ISRCTN and PubMed, we used “[drug name] AND cancer AND (radiation therapy OR radiotherapy)”. We then analysed the trial descriptions manually—only trials investigating drugs proposed to have radiosensitising activity (whether outlined on their trial rationale or, if unclear, drugs known to have preclinical evidence of radiosensitising effects on PubMed) were considered for this review (online supplemental table 1). The relevant data (drug, patient number, main indication, phase, trial identifier, cancer type, trial description, status and results) were extracted manually and summarised on a Word document contemporaneously. The list was then alphabetically ordered according to drug name (online supplemental table 1). A second check against the inclusion criteria was performed to ensure all drugs included on online supplemental table 1 have non-cancer indications, proposed to have radiosensitising activity, and have been investigated in clinical trials. Duplication of extraction by a second author was not performed, however, additional checks on selected drugs with higher volume of clinical trials were carried out by all authors to avoid omission of trials.

10.1136/bmjonc-2023-000192.supp1 Supplementary data

## A summary of the clinical trials to date

**Figure 2:**
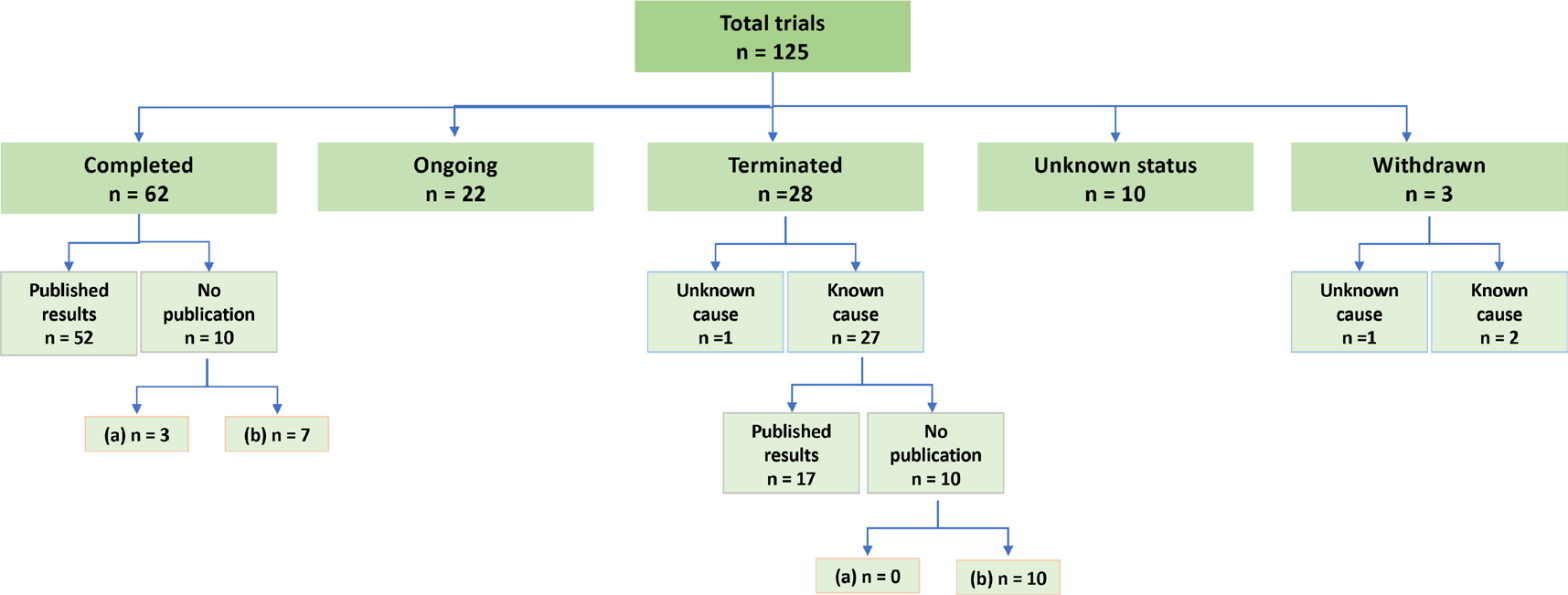
A summary of the clinical trials that have tested non cancer drugs as radiosensitisers. For the trials with published results, both meeting abstracts and research articles were included. ‘Unknown status’ defines a trial whose last known status was recruiting; not yet recruiting; or active, not recruiting, but that has passed its completion date, and the status has not been last verified within the past 2 years. The number of the trials with no published results which have been completed or terminated less (a) or more (b) than 2 years ago are indicated.

A total of 42 drugs were identified from the above methodology (online supplemental table 1). Thirteen drugs have only been studied in phase I trials, 19 drugs in phase II trials and only 10 drugs have progressed to phase III trials. Two drugs, nicotinamide and nimorazole, have been successfully repurposed. As of August 2023, of the 125 trials identified (online supplemental table 1, figure 2), 62 have been completed, 22 are still ongoing, 28 trials were terminated prematurely, 10 trials had unknown status and 3 trials were withdrawn before any patient enrolment. Sixty-nine trials reported their results, seven of which were in meeting abstracts only. We did not find any results published, either in abstract or research article, for 21 of the trials recorded as either ‘completed’ (n=10) or ‘terminated’ (n=11) on the registers. Slow accrual (n=11) is the most common cause of termination. Five trials were terminated due to loss of funding (metformin, n=2; nelfinavir, n=1; paricalcitol, n=1; and sirolimus, n=1). Others were stopped due to safety concerns (n=3), drug unavailability (n=1) or following futility analysis (n=6). The main indications of these 42 drugs are varied, with antimicrobials being the most common class (online supplemental table 1).

## Mechanisms of action and identification of candidate drugs

**Figure 3:**
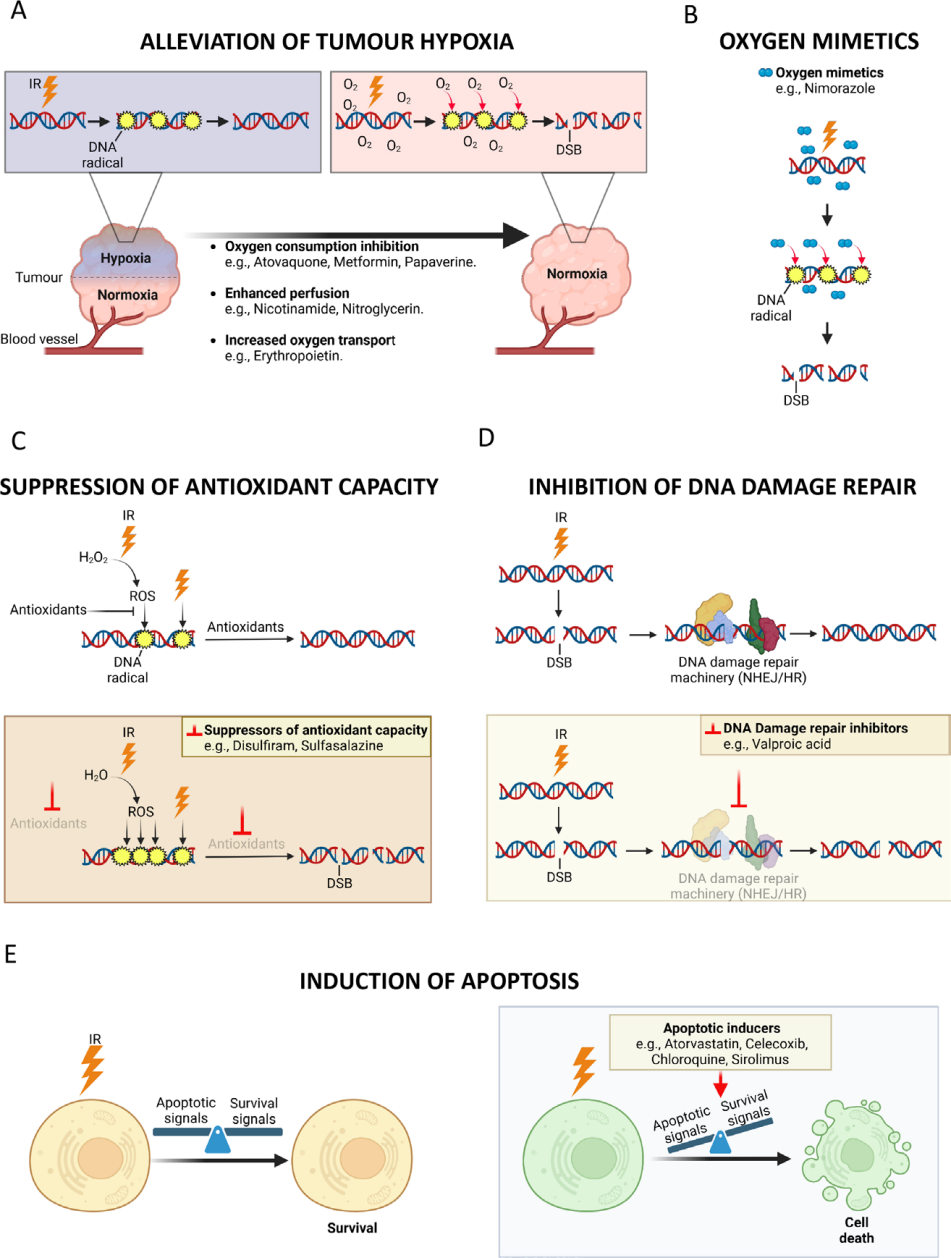
Mechanisms of action of drugs that have attempted to be repurposed as radiosensitisers. The figure illustrates the proposed mechanisms of action of non-cancer drugs that have been investigated clinically as radiosensitisers. Some examples of these drugs are provided. (A) Hypoxic cells can be up to three times more resistant to ionising radiation (IR) than normoxic cells. Therefore, alleviating tumour hypoxia is a suitable strategy to improve the efficacy of radiotherapy (RT). IR ionises the DNA molecule producing DNA radicals. According to the ‘oxygen fixation’ hypothesis, in the absence of oxygen, these DNA radicals are quickly reduced to its original state. When oxygen is present, conversely, the oxygen molecules react with the DNA radicals, thus stabilising (‘fixing’) the DNA lesion in the form of double-strand breaks (DSBs), which are potentially lethal for the cell. Hypoxic tumours can theoretically be oxygenated through different pharmacological approaches, including the inhibition of oxygen consumption in tumour cells, or enhancing tumour perfusion or oxygen transport in blood.^90 91^ (B) Based on the same principle of the ‘oxygen fixation’ hypothesis, drugs that mimic oxygen to ‘fix’ the IR-induced DNA damage can potentially be used to enhance radiosensitivity, especially under hypoxia.^91^ (C) The redox metabolism is a key determinant of radiosensitivity. The induction of DNA radicals by IR occurs both directly and indirectly through the generation of reactive oxygen species (ROS)—like the hydroxyl radical derived from the hydrolysis of water, considered the most cytotoxic mediator of the indirect effects of IR. The reduction of both IR-induced ROS and DNA radicals carried out by cell antioxidants—for example, ROS-detoxifying enzymes, sulfhydryl-containing molecules—contribute to diminish the cell radiosensitivity. Accordingly, targeting the antioxidant capacity of tumour cells can be leveraged to enhance their radiosensitivity.^90 92^ (D) Cells possess different molecular mechanisms to repair IR-induced DSBs, of which the non-homologous end joining (NHEJ) and the homologous recombination (HR) repair pathways are the main ones. The inhibition of the DNA machinery results in the persistence or mis-repair of IR-induced DSBs, which leads to chromosomal aberrations and ultimately to cell death, typically by mitotic catastrophe.^93–95^ (E) Cells subjected to IR can also undergo apoptosis, a type of programmed cell death which, unlike mitotic catastrophe, occurs in interphase and relatively early after IR exposure. The initiation of the apoptotic programme in the cell depends on the balance between apoptotic and survival signals, some of which stem from the DNA repair machinery and the redox status of the cell. These survival/apoptotic mediators—as well as the DNA repair pathways or the redox metabolism—can be targeted to favour the induction of apoptosis in response to IR.^93 96^ The inhibition of autophagy—a process of self-degradation of cell organelles, considered in certain contexts a cell survival mechanism—can also induce apoptosis in response to RT.^69–71^

The proposed mechanisms of action (MOA) through which the drugs listed in online supplemental table 1 exert their radiosensitising effect are varied, and include the alleviation of hypoxia, the fixation of DNA damage (oxygen mimetics), the suppression of antioxidant capacity, the inhibition of DNA damage repair and the induction of apoptosis. The fundamentals of these MOA with some drug examples are illustrated in figure 3. For some of these drugs, their potential radiosensitising capacity has been inferred from the existing knowledge on the MOA pertaining to the original indication. For instance, erythropoietin (EPO) was known to increase oxygen transportation and hence it was hypothesised that EPO could reduce tumour hypoxia, thus increasing radiosensitivity.^9^ Another example is nitroglycerin and its proposed role in reducing hypoxia-mediated radioresistance through its known vasodilator effect.^10 11^ Other drugs were discovered to exert a new MOA irrelevant for the non-cancer indication, but which suggested their utility as radiosensitisers. This is the case of valproic acid which, independently of its anticonvulsant activity, was found to inhibit histone deacetylase, a regulator of chromatin compaction involved in DNA damage repair and the response to ionising radiation.^12 13^ Candidate compounds can also be identified via high-throughput screening (HTS). Thus, atovaquone was shown to inhibit oxygen consumption and subsequently alleviate tumour hypoxia by means of an HTS of Food and Drug Administration (FDA)-approved drugs.^14^ Additionally, both prospective and retrospective observational studies where patients who received RT were incidentally treated with a certain non-cancer medication have also contributed to the identification of candidate drugs. Thus, for instance, the administration of aspirin,^15^ metformin^16–18^ or statins^19 20^ during RT was associated with better outcomes in observational studies, which have motivated the clinical testing of these non-cancer drugs in combination with RT.

## Examples of successful and unsuccessful repurposing efforts

This section highlights selected drugs and trials from online supplemental table 1. First, we discuss past trials and current states of the two successfully repurposed drugs, nicotinamide and nimorazole (table 1). This is followed by our interpretation of why once promising candidates have failed to pass the scrutiny of large randomised trials, taking as examples three drugs that have been subjected to intensive clinical research—metformin, celecoxib and EPO (table 2). Lastly, we summarise the fate of three drugs that have produced mixed results—nitroglycerin, chloroquine and hydroxychloroquine (table 3).

**Table 1:** Successfully repurposed drugs as discussed in section 3.1

Drugs	Main indication(s)	Phase(s)	Trial identifier	Cancer type(s)	Trial description	Patient numbers	Status/results (as per August 2023)
Nicotinamide	Treatment for niacin deficiency	2	–	Bladder cancer	Three-arm phase II trial involving patients with bladder cancer who would be included in one of three arms—carbogen, nicotinamide, or both carbogen and nicotinamide—while receiving radical RT	Enrolled: 30	Completed. Results: Well tolerated in all three groups. Favourable rates of local control at 6 months. (DOI: 10.1038/bjc.1997.372)
			–	GBM	Two-arm phase II trial evaluating the toxicity and efficacy of RT and chemotherapy (carmustine) with or without carbogen and nicotinamide in inoperable biopsy-proven GBM	Enrolled: 33	Completed. Results: Carbogen and nicotinamide only tolerated in half of patients in treatment arm. No significant differences in OS between treatment groups (DOI: 10.1016 /s0167-8140 (03)00007 – 0)
			ISRCTN08912168; PROCON	Prostate cancer	Single-arm phase II trial of RT in conjunction with carbogen and nicotinamide (CON) in prostate cancer	Enrolled: 50	Completed. Results (abstract summary only, no peer-reviewed publication available): Well tolerated (https://christie.openrepository.com/handle/10541/624399)
		3	NCT00033436	Bladder cancer	Randomised phase III trial to compare the effectiveness of RT with or without carbogen and niacinamide (CON) in patients with locally advanced bladder cancer.	Enrolled: 333	Completed. Results: CON+RT showed nonsignificant improvement in cystoscopic control at 6 months compared with RT alone (DOI: 10.1200/JCO.2010.28.4950)
			NCT00147732	Laryngeal cancer	Randomised phase III clinical trial comparing accelerated radiotherapy (AR) with AR plus carbogen and nicotinamide (ARCON) in clinical stage T2-4 laryngeal carcinoma.	Enrolled: 345	Completed. Results: ARCON showed no significant improvement in local tumour control (primary endpoint) compared with AR. (DOI: 10.1200/JCO.2011.35.9315)
Nimorazole	Antifungal	1	–	HNSCC	Phase I study of nimorazole in patients with HNSCC	Enrolled: 17	Completed. Results: Well tolerated (DOI: 10.1016/0360-3016(84 )90545-5)
		2	DAHANCA 18	HNSCC	Single-arm phase II trial on locally advanced head and neck cancer treated with AR, nimorazole and weekly cisplatin	Enrolled: 227	Completed. Results: Well tolerated. Better locoregional control (primary endpoint) of 80% compared with historical DAHANCA data (70%) (DOI: 10.3109/0284186 X .2014.992547)
			DAHANCA 28	HNSCC	Phase I/II feasibility study of hyperfractionated, AR with concomitant cisplatin and nimorazole (HART-CN) for patients with locally advanced, HPV/p16-negative squamous cell carcinoma of the oropharynx, hypopharynx, larynx and oral cavity	Enrolled: 50	Completed. Results: Combined treatment was feasible but was associated with significant acute toxicities. (DOI: 10.1016 /j .radonc.2020.03.025)
			–	HNSCC	Single-arm phase II trial to determine efficacy of hyperfractionated accelerated radiation therapy (CHART)/nimorazole regimen.	Enrolled: 61	Completed. Results: Local control rates in CHART+nimorazole were better than those previously seen with CHART alone (DOI: 10.1016 /s0167-8140 (02)00284 – 0)
			CTRI/2019/02/017477	Cervical cancer	Randomised, two-arm phase II trial of nimorazole with CRT for LACC compared with CRT alone	Target: 196	Status unknown. (Last updated 22 November 2019 on trial register CTRI)
		3	DAHANCA protocol 5–85	HNSCC	Randomised, placebo-controlled, double-blind phase III study by the Danish Head and Neck Cancer Study (DAHANCA) on nimorazole with RT in supraglottic larynx and pharynx carcinoma	Enrolled: 422	Completed. Results: Statistically significant improvement in locoregional control (primary endpoint) in those who received nimorazole compared with placebo. (DOI: 10.1016 /s0167-8140 (97 )00 220 x)
			NCT01950689; NIMRAD	HNSCC	Randomised, placebo-controlled trial of nimorazole and RT vs RT alone in patients with locally advanced HNSCC unsuitable for synchronous chemotherapy or cetuximab	Enrolled: 338	Completed. Results (abstract summary only, no peer-reviewed publication available): Addition of nimorazole did not improve locoregional control or survival compared with placebo (DOI: 10.1200/JCO.2023.41.16_suppl.6006)
			NCT01507467; IAEA-HypoX	HNSCC	Randomised, two-arm phase III trial of accelerated RT with or without nimorazole in HNSCC	Enrolled: 104 (Target: 600)	Terminated (Slow accrual) Results: Inconclusive as underpowered; improvements in locoregional tumour control and OS with the addition of nimorazole did not reach statistical significance. (DOI: 10.1016 /j .radonc.2015.04.005)
			NCT01880359; EORTC-1219	HNSCC	Randomised, placebo-controlled phase III trial of accelerated fractionated CRT with or without nimorazole, using a 15-gene signature for hypoxia in the treatment of HNSCC	Target: 640	Status unknown. Estimated completion time: January 2023 (Last updated 10 May 2022 on ClinicalTrials.gov)
			DAHANCA30; NCT02661152	HNSCC	Randomised non-inferiority phase III trial of hypoxia-profile guided hypoxic modification of RT with nimorazole in patients with HNSCC	Target: 1252	Ongoing. Estimated study completion: December 2023.

CRT, chemoradiotherapy; HNSCC, head and neck squamous cell carcinoma.

**Table 2:** Examples of failed attempts at repurposing as discussed in section titled, “Failed attempts at repurposing”

Drugs	Main indication(s)	Phase(s)	Trial identifier	Cancer type(s)	Trial description	Patient numbers	Status/Results (as per August 2023)
Metformin	Hypoglycaemic	1	NCT02149459	Brain tumour	Phase I trial studying toxicity profile and compliance of metformin, moderately low carbohydrate diet and RT	Target: 18	Status unknown. (Last updated 27 October 2017 on ClinicalTrials.gov)
			NCT02153450	Pancreatic cancer	Open-label pilot, single-centre, non-randomised phase I trial to assess tolerability and preliminary activity of the combination of stereotactic body radiation therapy (SBRT) with metformin for resectable and locally advanced pancreatic/periampullary cancers	Enrolled: 8	Completed. Results unknown. (Last updated 26 June 2020 on ClinicalTrials.gov)
		1&2	NCT02949700	HNSCC	Single-arm phase I/II trial of metformin in combination with cisplatin and RT in HNSCC	Enrolled: 26	Completed. Results unknown. (Last updated 29 June 2023 on ClinicalTrials.gov)
			NCT04536805; REPAIRGETUGP16	Prostate cancer	Randomised, three-arm trial to study safety and efficacy of adding metformin to stereotactic ablative reirradiation to patients with relapse in previously irradiated prostate bed	Target: 46	Recruiting. Estimated study completion year: 2028
		2	NCT02945813; SAKK 08/15 - PROMET	Prostate cancer	Randomised, two-arm phase II trial of salvage RT with or without metformin in non-diabetic patients with prostate cancer after prostatectomy	Enrolled: 111 (Target: 170)	Terminated early by the SAKK Board due to financial reasons. Results (abstract summary only, no peer-reviewed publication available): No significant improvement in time to progression (TTP) in arm treated with metformin compared with control arm. Trial was underpowered due to premature termination (DOI: 10.1200/JCO.2023.41.6_suppl.353)
			NCT04275713; METOXY-LACC	Cervical cancer	Randomised, two-arm phase II trial studying altered tumour oxygenation in patients with LACC receiving cisplatin and RT with or without metformin Tumour oxygenation will be evaluated by gene signatures and MRI parameters.	Target: 90	Recruiting. Estimated study completion year: 2025.
			NCT02394652	Cervical cancer	Randomised, two-arm phase II trial evaluating tumour oxygenation in patients with LACC receiving radical RT and concurrent cisplatin chemotherapy with or without metformin.	Enrolled: 20	Completed. Results: Reduction in tumour hypoxia as evaluated on FAZA-PET scan (DOI: 10.1158/1078 – 0432 .CCR-22 – 1665)
			NCT04170959; RADFORMIN	NSCLC	Observational lead-in phase I and a randomised, three-arm phase II trial studying the addition of metformin to definitive RT in patients with inoperable stage III NSCLC	Enrolled: 3	Terminated (Loss of external funding)
			NCT02285855	NSCLC	Randomised, two-arm, placebo-controlled phase II trial evaluating the addition of metformin to SBRT in NSCLC	Enrolled: 27 (Target: 70)	Terminated (Slow accrual)
			NCT02473094; NEOMETRE	Rectal cancer	Randomised, two-arm, placebo-controlled phase II trial to evaluate the efficacy and tolerability of metformin in addition to CRT for the preoperative locally advanced rectal carcinomas.	Enrolled: 3 (Target: 98)	Terminated (Slow accrual)
			NCT02186847; NRG-LU001	NSCLC	Randomised, two-arm phase II trial investigating outcomes in patients with stage III NSCLC who received either CRT (carboplatin-paclitaxel and RT) alone or CRT with metformin	Enrolled: 170	Completed. Results: Worse PFS at 1 year (primary endpoint) in treatment arm. (DOI: 10.1001/jamaoncol.2021.2318)
			NCT02115464; OCOG-ALMERA	NSCLC	Randomised, open-label, two-arm phase II trial evaluating outcomes in NSCLC patients receiving either CRT (cisplatin-based regimen) alone or CRT and metformin	Enrolled: 54 (Target: 96)	Terminated (Slow accrual) Results: Addition of metformin to CRT was associated with worse outcomes (PFS at 1 year and OS) and increased toxicities compared with CRT alone. (DOI: 10.1001/jamaoncol.2021.2328)
Celecoxib	Anti-inflammatory	1	NCT00177853	Pancreatic cancer	Single arm phase I study to investigate safety and efficacy of celecoxib with irinotecan and concurrent RT in preoperative pancreatic cancer	Target: 23	Terminated (Unknown cause)
			–	Nasopharyngeal	Phase I trial investigating the safety of concurrent celecoxib and RT in locoregionally advanced nasopharyngeal carcinoma	Enrolled: 34	Completed. Results: Well tolerated. (DOI: 10.1016 /j .oraloncology.2011.06.002)
			–	Unresectable NSCLC	Phase I clinical trial of thoracic RT and concurrent celecoxib for inoperable/unresectable NSCLC	Enrolled: 47	Completed. Results: Well tolerated at FDA-approved dose. (DOI: 10.1158/1078 – 0432 .CCR-04 – 1741)
		1&2	–	Oesophageal cancer	Phase 1/II trial assessing the MTD of celexocib combined with CRT (cisplatin, irinotecan and RT) in locally advanced oesophageal cancer	Enrolled: 13 (before termination)	Terminated (External safety concerns - for example, cardiovascular toxicities and thromboembolic risks - from the FDA and Therapeutic Goods Administration (Australia)) Results: Primary endpoint (MTD) not reached. (DOI: 10.1007 /s10637-006-9016-5)
		2	NCT00181532	NSCLC	Randomised, double-blinded placebo-controlled phase II trial to evaluate tumour response and toxicity profile in celecoxib and concurrent RT in stages II–III NSCLC	Enrolled: 40 (target of 102)	Terminated (Slow accrual) Results: Formal statistical analysis not performed. HR is 0.62 in favour of celecoxib arm for OS. (DOI:https://doi.org/10.1016/j.radonc.2007.05.008)
			NCT00520091	Oesophageal Cancer	Non-randomised two-arm phase II trial of irinotecan, cisplatin and RT with or without celecoxib in stages II–IV oesophageal cancer	Target: 14	Completed. Results unknown. (Last updated 18 May 2012 on ClinicalTrial.gov)
			NCT00137852	Oesophageal Cancer	Non-randomised, single-arm phase two trial studying the response rate and safety of adding celecoxib to neoadjuvant cisplatin-irinotecan chemoradiation for operable oesophageal cancer	Enrolled: 40	Completed. Result: While the addition of celecoxib was tolerable, OS was comparable to using neoadjuvant cisplatin-irinotecan chemoradiation alone in historical studies (DOI: 10.1186 /s12885-016-2485-9)
			JPRN-UMIN000012818	Locally advanced rectal cancer	Non-randomised, single**-**arm, single centre phase I/II trial studying MTD and efficacy of preoperative CRT using S-1 combined with celecoxib for advanced lower rectal cancer	Enrolled: 21	Completed. Results: Primary endpoint of 30% target pathological complete remission (pCR) rate was not met (15.8%), indicating no synergistic or additive effects. (DOI: 10.23922/jarc.2018 – 026)
			NCT00068770	Glioblastoma	Non-randomised, two-arm phase II trial studying the effects of hepatic enzyme-inducing antiseizure drugs (+EIASD) on the pharmacokinetics of celecoxib in patients with newly diagnosed GBM receiving RT. Those received EIASD (+EIASD) were compared against those who did not (−EIASD).	Enrolled: 35	Terminated after the EORTC trial (DOI: 10.1056/NEJMoa043330) showed temozolomide (TMZ) and RT conferred significant survival in this population). Results: Well tolerated. No differences between pharmcokinetic parameter between the groups (DOI: 10.1215/15228517-2007-055)
			NCT01503385	Unresectable NSCLC	Single-centre, open-label, randomised phase II trial of cisplatin/etoposide and concurrent RT wth or without celecoxib in patients with unresectable locally advanced NSCLC	Enrolled: 100	Completed. Results: No improvement in OS (primary endpoint). (DOI: 10.1001/jamanetworkopen.2019.18070
			–	NSCLC	Phase II study of celecoxib in combination with paclitaxel, carboplatin and RT for patients with inoperable stage IIIA/B NSCLC	Enrolled: 17	Terminated because predetermined goal of 80% overall response rate was not met. Results: No improvement in survival. (DOI: 10.1158/1078 – 0432 .CCR-08 – 0629)
			–	Pancreatic cancer	Single-arm phase II trial investigating toxicity and efficacy of short intensive uracil/tegafur based CRT combined with celecoxib in locally advanced pancreatic cancer	Enrolled: 83.	Completed. Results: Poorly tolerated; no partial or complete response observed; poor survival consistent with other studies with 5-fluouracil and CRT (DOI: 10.1016 /j .radonc.2010.10.016)
			–	Rectal cancer	Single-arm phase II trial investigating the effects of celecoxib combined with preoperative chemoradiation (uracil-tegafur and RT) for locally advanced rectal cancer	Enrolled: 35	Completed. Results: Complete pathological remission (primary endpoint) target of>15% was met. High incidence (49%) of rash that led to non-compliance. (DOI: 10.1007 /s00384-007-0407-7)
Erythropoietin (EPO)	Treatment for anaemia secondary to chronic kidney disease	3	RTOG 99 – 03	HNSCC	Randomised controlled phase III trial by the Radiation Therapy Oncology Group (RTOG) investigating the outcomes of anaemic patients with HNSCC receiving RT with or without EPO (epoetin)	Enrolled: 148 (Target: 372)	Terminated (interim analysis showed futility). Results: Non-statistically significant trend towards worse locoregional failure rate in EPO arm. (DOI: 10.1016 /j .ijrobp.2007.04.063)
			–	HNSCC	Randomised, double-blind, placebo-controlled phase III trial on the addition of EPO to treat HNSCC patients with anaemia undergoing RT	Enrolled: 351	Completed. Results: Epoetin β is associated with statistically significant worse locoregional PFS (primary endpoint) compared with placebo. (DOI: https://doi.org/10.1016/S0140-6736(03)14567–9)
			NCT00348738	Cervical cancer	Randomised controlled phase III trial on disease-specific survival, tumour response and local control in patients with cervical cancer who receive definitive RT with or without EPO	Target: 300	Status unknown. (Last updated 2 November 2007 on ClinicalTrials.gov)
			NCT00017004	Cervical cancer	Randomised phase III trial studying the efficacy of maintaining haemoglobin level above 120 g/L with EPO vs above 100 g/L without EPO in anaemic patients receiving concurrent RT and cisplatin for cervical cancer	Enrolled: 114 (< 25% of target of 460)	Terminated (Potential concern by study sponsor regarding thromboembolic event with study drug) Results: Inconclusive (DOI: 10.1016 /j.ygyno.2007.10.011
			–	HNSCC	Randomised open-label phase III trial evaluating effects of epoetin alfa on local disease-free survival in patients receiving RT with curative intent	Enrolled: 301	Completed. Results: No benefit in survival with the addition of epoetin alfa to RT compared with RT alone. (DOI: 10.1200/JCO.2009.22.3693)

CRT, chemoradiotherapy; FDA, Food and Drug Administration; HNSCC, head and neck squamous cell carcinoma; MTD, maximum tolerated dose; NSCLC, non-small cell lung cancer; PFS, progression-free survival; RT, radiotherapy.

**Table 3:** Examples of candidates with mixed results as discussed in section titled, “Candidates with mixed results”.

Drugs	Main indication(s)	Phase(s)	Trial identifier	Cancer type(s)	Trial description	Patient numbers	Status/results (as per August 2023)
Nitroglycerin	Vasodilator	1	NCT01407107	Rectal cancer	Phase I dose-escalation trial of nitroglycerin in addition to 5-fluorouracil and RT for neo-adjuvant treatment of operable rectal cancer	Enrolled: 13	Completed. Results: Well tolerated (DOI: 10.1016 /j .surg.2015.04.007)
		2	NCT04338867	Brain metastases in NSCLC	Randomised controlled, open-label phase II trial evaluating the addition of nitroglycerin to whole intracranial RT for brain metastases in NSCLC	Enrolled: 96	Completed. Results: Treatment arm had better intracranial objective response rate (iORR) and intracranial PFS compared with control arm. (DOI: 10.1016 /j .ijrobp.2022.02.010)
			NCT00886405	NSCLC	Single-arm phase II study with concurrent CRT (vinorelbine+cisplatin) with nitroglycerin for locally advanced NSCLC	Enrolled: 35	Completed. Results: Well tolerated. OS was better (54%) than historical data (30%) from SWOG 8805 (DOI: 10.1016 /j .radonc.2014.01.021)
			NCT01210378	NSCLC	Single-arm phase II trial evaluating 2-year overall survival (OS) (primary endpoint) in stages IB–IV NSCLC patients treated with radical (chemo-) RT and nitroglycerin patch during RT	Stopped at 42	Terminated (Accrual stopped following futility analysis—no reduction in tumour hypoxia on serial hypoxia positron emission tomography/computed tomography (PET/CT scans)) (DOI: 10.1016 /j .ctro.2019.12.002)
Chloroquine	Antimalarial	1	NCT01727531	Brain metastases	Single-arm study to evaluate efficacy of adding chloroquine to whole brain RT in patients with brain metastases and whether the IDO2 genetic status informs efficacy of his combination	Enrolled: 20	Completed. Results (abstract summary only, no peer-reviewed publication available): Inconclusive (DOI:https://doi.org/10.1016/j.ijrobp.2011.06.1313)
			NCT02378532; CHLOROBRAIN	Glioblastoma	Single-centre, open-label, dose-finding phase I trial for the addition of chloroquine to temozolomide and concurrent RT in glioblastoma	Enrolled: 13	Completed. Results: Well tolerated, MTD established. (DOI: 10.1080/15548627.2020.1816343)
			NCT04397679	Glioblastoma	Open-label phase I trial for partial brain RT, temozolomide, chloroquine and tumour treating field therapy for newly diagnosed glioblastoma	Target: 10	Recruiting. Estimated study completion year: 2025 (Last updated 24 April 2023 on ClinicalTrials.gov)
			NCT00969306; Chloroquine IV	SCLC	Non-randomised phase I trial studying toxicity and response of adding chloroquine to cisplatin-etoposide in extensive disease SCLC or to concurrent RT and cisplatin-etoposide in limited disease SCLC	Enrolled: 5	Terminated (Slow accrual)
		2	NCT01894633	Brain Metastases	Randomised, double-blind, placebo-controlled phase II study of whole-brain irradiation with concomitant chloroquine for brain metastases	Enrolled: 73	Completed. Results: Chloroquine used concurrently with RT is well tolerated and improves local control and PFS compared with the control arm (DOI: 10.1186/1748 – 717 X-8-209)
			NCT02432417; CHLOROBRAINII	Glioblastoma	Randomised controlled phase II trial for the addition of chloroquine to CRT for glioblastoma with endpoint of PFS-6.	Target: 156	Estimated study completion year: 2025 (Last updated 12 April 2022 on ClinicalTrials.gov)
		3	NCT00224978	GBM	Randomised, placebo-controlled, double-blind phase III trial for the addition of chloroquine as adjuvant to conventional treatment for GBM	Enrolled: 30	Completed. Results: Improvement in median survival (primary endpoint) did not reach statistical significance (DOI: 10.7326/0003-4819-144-5-200603070-00008)
Hydroxychloroquine (HCQ)	Antimalarial; treatment for rheumatoid arthritis and SLE	1	NCT01417403	Solid tumours with bone metastases	Phase I trial to establish MTD of HCQ in patients with solid tumours undergoing RT for bone metastases	Enrolled: 10	Terminated (published data established safety of using higher dose than dose in current study—see DOI: 10.4161/auto.28984)
		1&2	NCT00486603	GBM	Dose-escalation phase I and non-comparative phase II trial to assess safety and efficacy of HCQ in conjunction with RT and concurrent and adjuvant TMZ in patients with newly diagnosed GBM.	Enrolled: 16 in phase I, 76 in phase II	Completed. Results: No significant improvement in OS when compared with cohort from EORTC trial (TMZ+RT vs RT alone) (DOI: 10.4161/auto.28984)
		2	NCT01602588	High grade gliomas (HGG)	Randomised, two-arm phase II trial investigating survival at 1 year with the addition of HCQ to short course RT (SCRT) in patients aged ≥70 years with HGG	Enrolled: 54	Terminated (as advised by the IDMC due to differences in survival) Results: Worse survival in HCQ arm compared with control arm. (DOI: 10.1093/noajnl/vdaa046)
			NCT04011410	Oligometastatic prostate cancer	Single-arm, non-blinded phase II trial to evaluate tumour suppressor PAR-4 levels from baseline in patients with oligometastatic prostate cancer treated with 3 months of HCQ in combination with RT or surgery	Target: 20	Ongoing. Estimated study completion year: 2026
			NCT01494155	Resectable pancreatic cancer	Single-arm phase II trial studying the efficacy of SCRT with proton or photon beam capecitabine and HCQ for resectable pancreatic cancer	Target: 50	Status unknown. Estimated study completion: January 2023. (Last updated 2 February 2021 on ClinicalTrials.gov)

CRT, chemoradiotherapy; NSCLC, non-small cell lung cancer; PFS, progression-free survival; RT, radiotherapy.

### Successfully repurposed drugs

Nicotinamide, or niacinamide, is used for various disorders, including pellagra and schizophrenia. Preclinical studies demonstrated nicotinamide’s potential in enhancing RT effects by increasing tumour blood perfusion and tumour oxygenation (figure 3).^21–23^ Preclinical studies in murine models highlighted the potential of nicotinamide in combination with carbogen (95% O_2_/5% CO_2_) to overcome acute and chronic hypoxia, respectively, and cooperatively enhance tumour radiosensitivity.^23 24^ A randomised phase III trial with 345 patients with laryngeal cancer observed a small difference in regional control with accelerated RT in combination with carbogen/nicotinamide compared with accelerated RT alone.^25^ However, improvement in local control, the primary endpoint, did not reach statistical significance.^25^ This contrasts with the more optimistic finding of the randomised phase III BCON (Bladder Carbogen Nicotinamide) trial (NCT00033436) involving 333 patients with bladder cancer—differences in overall survival (OS), death risk and local relapse were in favour of carbogen/nicotinamide combined with radical RT.^26^ Interestingly, results from the BCON trial found an association between the presence of tumour necrosis at diagnosis and favourable long-term outcomes after combined treatment with carbogen/nicotinamide and RT in patients with bladder cancer (5-year OS of 53% and 33% in patients with and without tumour necrosis, respectively).^27^ The assessment of tumour necrosis may, therefore, be useful to guide the selection of patients for treatment with carbogen/nicotinamide plus RT. Following the BCON trial, the United Kingdom’s National Institute of Care and Excellence guideline (NG2) recommends the use of radiosensitising treatments, such as carbogen/nicotinamide, alongside radical RT for patients with muscle invasive urothelial bladder cancer.^28^ However, many UK centres have elected to use chemotherapy agents as radiosensitisers instead, possibly because of fewer practical considerations associated with their use.

An alternative approach to enhance tumour radiosensitivity is with the use of oxygen mimetics (figure 3), such as the antibiotic nimorazole. In Denmark, nimorazole has been repurposed as a hypoxia-activated prodrug to radiosensitise head and neck squamous cell carcinoma (HNSCC).^29^ The randomised phase III DAHANCA-5 trial involving 422 HNSCC patients undergoing RT demonstrated better locoregional control in nimorazole-treated patients compared with placebo (5-year actuarial rate of 49% vs 33%, p<0.002).^30^ Despite being widely used in Denmark, nimorazole is not routinely used elsewhere. NIMRAD (NCT01950689) is a large UK multicentre phase III trial that randomised HNSCC patients unsuitable for concurrent platinum chemotherapy or cetuximab with definitive RT into receiving RT with either nimorazole or placebo.^31^ The results of this trial have been recently presented in a meeting abstract, and report no improvement in locoregional control or survival with the addition of nimorazole to RT.^31^ This is likely to further limit the potential of nimorazole to become an established treatment in combination with RT.

### Failed attempts at repurposing

Metformin is an antiglycaemic drug whose use in diabetic patients has been associated with improved outcomes after RT or chemoradiotherapy (CRT) in retrospective studies.^16–18^ One of the key mechanisms by which metformin purportedly exerts its radiosensitising effect is via inhibition of oxygen consumption in tumour cells and, ultimately, hypoxia reduction (figure 3).^14 32 33^ Despite promising results from preclinical^32 33^ and retrospective clinical studies,^16–18^ several prospective clinical trials that evaluated metformin in combination with RT/CRT yielded disappointing outcomes (table 2). Most notable are the NRG-LU001^34^ and the OCOG-ALMERA^35^ randomised phase II trials. These two studies investigated the addition of metformin given with concurrent CRT to non-diabetic patients with locally advanced NSCLC. NRG-LU001 found that metformin does not improve OS, and OCOG-ALMERA reported worse treatment efficacy and enhanced toxicity with the addition of metformin.^34 35^ A notable difference between these trials is that NRG-LU001 used paclitaxel plus carboplatin as chemotherapy, while OCOG-ALMERA used cisplatin. It was accordingly speculated that metformin could be toxic when combined with cisplatin-based CRT. Interruption of chemotherapy due to this toxicity may explain the worse outcomes in the metformin-treated group in OCOG-ALMERA.^35^ Another phase II trial that evaluated the capacity of metformin to alleviate tumour hypoxia in cervical cancer showed a relatively modest decrease in tumour hypoxia with metformin,^36^ suggesting that insufficient potency to target mitochondrial complex I in the tumour might partly explain the lack of efficacy of metformin in the OCOG-ALMERA and NRG-LU001 studies.

Cyclooxygenase 2 (COX2) expression in human tumours has been linked to radioresistance, while its inhibition was shown preclinically to increase tumour radiosensitivity through different MOA, including angiogenesis suppression and apoptosis induction (figure 3).^37–41^ These findings led to the clinical testing of celecoxib, a selective COX-2 inhibitor used as analgesia, concurrently with RT/CRT. However, none of the phase II trials demonstrated substantial survival benefit (table 2), including a two-arm randomised study that compared CRT alone (OS=32.8 months) vs celecoxib and CRT (OS=35.5 months), and which attempted to stratify NSCLC patients according to a surrogate marker of tumour COX-2 activity/expression.^42^ Of note, a single-arm phase II study evaluating celecoxib with CRT in NSCLC correlated the absence of response with high pretreatment levels of urine prostaglandin E_2_, a product of COX-2, and/or the inefficiency of celecoxib to reduce the levels of this marker.^43^ This suggests that celecoxib is not potent enough to produce a therapeutic degree of COX-2 inhibition in the tumour, especially in patients with high COX-2 expression/activity, who display more aggressive disease and reduced control after therapy.^42 43^ In 2005, the FDA warned about potential risks of cardiovascular toxicity with celecoxib.^44^ Consequently, a trial investigating celecoxib with CRT during that time was terminated.^45^ No other RT trials reported cardiovascular-related issues attributable to celecoxib (table 2). In addition to the lack of benefit, the reporting of non-cardiovascular toxicity and safety issues for some tested indications—like the combination of celecoxib with uracil/tegafur-based CRT in locally advanced pancreatic^46^ or rectal^47^ cancer—has also contributed to dismissing the repurposing of celecoxib as a radiosensitiser.

Another example of a non-cancer drug that has failed clinical testing in combination with RT is EPO. The rationale for administering EPO to RT-treated patients was to increase haemoglobin (Hb) levels and prevent anaemia, which is linked to adverse outcomes post-RT/CRT.^48–51^ It was proposed that tumours of anaemic patients are more radioresistant due to impaired oxygenation,^52^ and preclinical studies supported the utility of EPO to alleviate tumour hypoxia and enhance tumour radiosensitivity (figure 3).^53–56^ However, three randomised phase III trials comparing RT and EPO versus RT alone in HNSCC showed no benefit in local disease-free or OS,^57^ a trend towards worse locoregional failure rates (36% vs 44%)^58^ or significantly worse locoregional progression-free survival (PFS) (adjusted relative risk=1.62)^9^ with the addition of EPO, respectively. The exact cause for the adverse outcomes reported in EPO-treated patients is not clear, but has been attributed to (1) the overactivation of proliferative and antiapoptotic signalling by EPO in tumours expressing the EPO receptor^59^ and (2) the high levels of Hb achieved in blood, which paradoxically could have impaired tumour oxygenation and reduce radiosensitivity.^9 60^ EPO administration was associated with a higher incidence of thrombotic events, but this phenomenon was not consistently observed across different studies and seems independent of RT.^9 58 61–65^ Furthermore, the randomised controlled phase III DAHANCA-10 trial with a different erythropoiesis-stimulating agent, darbepoetin alfa, showed worse locoregional control in HNSCC patients treated with the drug plus RT compared with RT alone. Collectively, these negative outcomes in EPO trials have clearly shown that further attempts at repurposing them as radiosensitisers would be inappropriate.

### Candidates with mixed results

Nitroglycerin, a nitric oxide donor agent, is a vasodilator proposed to alleviate tumour hypoxia by improving tumour perfusion (figure 3).^10 11^ Several phase I and II trials investigating nitroglycerin and RT produced mixed results (table 3).^11 66–68^ Most notably, a phase II trial evaluating nitroglycerin and RT in NSCLC, nitroglycerin was found not to reduce hypoxia on serial HX4-hypoxia positron emission tomography/computed tomography (PET/CT scans).^11^ Another phase II trial, however, showed that concurrent whole brain RT (WBRT) and nitroglycerin in patients with brain metastases (BM) from NSCLC resulted in a better intracranial objective response rate compared with WBRT alone.^68^ A high proportion of NSCLC patients with BMs have the epidermal growth factor mutation (EGFRm) subtype, which is associated with increased risks of BM.^68^ Within the EGFRm subpopulation in the trial, a significant improvement in intracranial PFS was observed in nitroglycerin and WBRT compared with WBRT alone (27.7 vs 10.0 months).^68^ This finding suggests that future clinical trials testing nitroglycerin might benefit from preselecting patients with EGFRm.

While better known as an antimalarial, chloroquine has been evaluated as a radiosensitiser as evidence of its capacity to improve tumour perfusion or induce apoptosis through autophagy inhibition emerges (figure 3).^69–72^ Chloroquine has shown promising results in a placebo-controlled phase II trial, where its concomitant administration with WBRT in patients with BM from solid tumours resulted in better 1-year PFS (83.9%) compared with control (55.1%).^73^ Chloroquine has also been investigated clinically combined with CRT in glioma, a cancer type where autophagy is thought to play a key role in the resistance to RT and temozolomide.^70 74–76^ In this regard, the phase I CHLOROBRAIN trial in glioblastoma established the feasibility of coadministrating chloroquine with RT and temozolomide.^77^ This trial also evidenced a potential survival benefit with the addition of chloroquine to RT/temozolomide, especially in a subgroup of patients with a specific mutation in EGFR (EGFRvIII) linked to poor prognosis.^77^ This will be further explored in an ongoing, two-arm phase II trial (CHLOROBRAIN II; NCT02432417), alongside long-term outcomes. It should be noted, however, that a recent randomised phase II trial in high-grade glioma testing hydroxychloroquine, another autophagy inhibitor, reported worse 1-year OS in patients treated with hydroxychloroquine and RT compared with RT alone (41.2% vs 20.3 %).^78^ Although encouraging, the positive results seen when combining chloroquine and RT/CRT must be taken with caution, in view of the negative outcomes reported with the mechanistically and structurally related hydroxychloroquine.

## Challenges and future directions

On reviewing the trials, we identified several challenges in repurposing which unsurprisingly overlap with those of conventional drug development. However, an additional barrier to the former is the lack of financial support further compounded by patent issues. The following subsections discuss these challenges alongside suggestions for future repurposing efforts.

### Poor reporting of trial results

Strikingly, about one-quarter of the clinical trials we identified (30 out of 125) were terminated or withdrawn for unknown reasons (n=2), or had not updated their status (n=10) or published any results (n=18) within 2 years after the last trial register update or completion/termination, respectively (figure 2). Poor trial reporting is therefore a major barrier to understanding and proposing solutions to the challenges of repurposing drugs as radiosensitising treatments. The stricter implementation of policies on trial reporting and mechanisms to ensure compliance from research institutions and trial sponsors will hopefully mitigate this barrier.^79 80^

### Lack of biomarkers of response and patient selection

Biomarkers are useful tools to predict which patients are likely to benefit from treatment. Therefore, the use of appropriate biomarkers can be critical for the success of clinical testing. Thus, for example, as commented in section titled, “Candidates with mixed results”, the selection of patients based on the presence of mutations in EGFR might conceivably play an important role for the successful translation of nitroglycerin and chloroquine in combination with RT and RT/temozolomide, respectively.^68 77^ In addition, although only speculative, the exclusion of patients with tumours positive for the EPO receptor might have led to more favourable outcomes in the phase III trials that tested EPO in combination with RT/CRT.^59^ Notably, among the trials we identified that tested drugs that purportedly exert their radiosensitising effect via hypoxia alleviation, only a few selected/stratified patients by assessing tumour hypoxia.^11 36^ Tumour hypoxia can be reliably assessed clinically using hypoxia PET-CT, an imaging technique fully developed more than 15 years ago.^81^ However, its implementation has challenging practicalities and is expensive, which explains why it is not broadly used in clinical testing. The relatively recent development and validation of hypoxia gene-expression signatures provide an alternative and cheaper strategy for classifying patients according to hypoxia levels.^82 83^ Future trials testing radiosensitising drugs targeted to a specific group of patients should consider biomarker-guided patient selection. The discovery of robust biomarkers, and more practical and affordable methods of biomarker assessment will conceivably facilitate the repurposing of radiosensitising drugs.

### Lack or inappropriate consideration of evidence, suboptimal pharmacological properties and inadequate trial design

One advantage of drug repurposing is the availability of knowledge on pharmacological properties, which can be used to derisk, accelerate and reduce costs of clinical development. However, since the clinical context of the tested cancer application differs from that of the original indication, the ideal pharmacological properties required in such divergent scenarios may also differ.

Indeed, suboptimal pharmacological properties are a likely cause of failure in the repurposing of some radiosensitising drugs. Thus, for example, as commented in section titled “Failed attempts at repurposing”, suboptimal potency seems a plausible explanation for the lack of efficacy of metformin and celecoxib when combined with RT/CRT.^14 33 43^ Regarding metformin, it has long been known that at pharmacological concentrations it only produces a small decrease in oxygen consumption,^14 33^ which may explain the modest effect that metformin has in decreasing tumour hypoxia clinically.^36^ Based on the purported MOA, preclinical studies should address whether a drug has optimal pharmacokinetics and hits the corresponding target at clinically relevant concentrations, by using robust models and appropriate biomarkers. Furthermore, toxicity issues were reported for 5 of the 42 drugs we identified (celecoxib, metformin, nelfinavir, nicotinamide and nimorazole) (online supplemental table 1), despite that some of these drugs were tested at doses similar to those used in the original indication. Taking celecoxib as an example, the toxicity seems to occur when this drug is combined with uracil/tegafur-based CRT, but not with other CRT modalities or RT alone (online supplemental table 1). To the best of our knowledge, there are no preclinical studies testing the combination of celecoxib with uracil/tegafur, either alone or with RT, which might have potentially revealed the toxicities seen in the clinical trials. These examples highlight the unavailability or inadequate consideration of data as a possible limitation to judge whether a drug has optimal pharmacological properties.

The attempt to repurpose EPO as a radiosensitiser also exemplifies how the lack of evidence might determine the success of a clinical trial. The recruitment of patients with normal Hb levels and the relatively high Hb levels achieved in blood may lie behind the worse outcomes observed with the addition of EPO to RT in HNSCC patients.^9 58^ Unfortunately, the evidence indicating that increasing Hb above normal levels could be detrimental for tumour oxygenation—and consequently for the efficacy of RT—was published during or shortly after the completion of the corresponding randomised phase III trials,^9 57 58 60^ which could have otherwise been taken into consideration for optimal trial design (eg, recruiting anaemic patients and setting lower Hb target levels).

Obtaining and carefully considering relevant evidence, preclinical and clinical, is therefore needed to decide whether it is appropriate pursuing the clinical testing of a drug, for optimal trial design, and ultimately, to increase the likelihood of success in the repurposing of radiosensitising drugs.

### Lack of funding support

The financial support for repurposing trials plays an important role in their fate. Five of the trials we identified were terminated due to funding issues. Only two of these trials reported results, but due to premature termination one was underpowered to demonstrate benefit or the lack thereof,^84^ and the other one failed to establish the maximum tolerated dose^85^ (online supplemental table 1). In another phase I trial in glioblastoma that successfully established the maximum tolerated dose and recommended phase II dose for dimethyl fumarate (DMF, used to treat psoriasis and multiple sclerosis), progression to later phases was impaired not by trial outcomes but rather the manufacturers’ decision not to further develop DMF for glioblastoma,^86^ and appears to have been decided by financial considerations. This highlights that with a lack of financial motivation to pursue these drugs, their RT combination may be jeopardised despite their clinical potential.

It cannot be easily ascertained how many non-cancer drugs with radiosensitising capacity have not been taken to clinical testing for combined treatment with RT because of financial reasons, but there are solid theoretical grounds to think that the lack of financial incentive is a major limitation for drug repurposing. The lack of financial incentive is often attributed to patent issues. Clinical trials for de novo drugs are usually sponsored by pharmaceutical companies looking for a return of investment (ROI) once the drug is patented and given market exclusivity.^6^ However, with generic drugs used for repurposing, the low ROI and the absence of a strong patent for these drugs mean that competing companies could capitalise on drug production following trial outcomes, hence diminishing the prospects of the company that funded the trial.^6^

To address some of these challenges, regulators such as the European Medicines Agency and the FDA have offered financial incentives such as granting data exclusivity and market protection for a new indication.^6^ In April 2023, the European Commission (EC) announced that the regulatory data protection would be for a minimum of 8 years. This could be extended in circumstances, including, but not limited to, if products are made available across EU member states or if a comparative trial is conducted.^87^ In the UK, the Medicines and Healthcare products Regulatory Agency offers a 1-year extension to the 10-year market protection period if authorisation for a new indication is gained during the first 8 years.^88^ As some of these implementations are relatively recent, the outcome of these incentives is not yet apparent. Alternatively, to address the profit-driven limitations of drug repurposing, collaborations between charity schemes, philanthropists and academics coupled with dedicated fundings for repurposing,^89^ could help ensure that repurposing remains one of the ways in which more patients have access to new therapeutic options. It is clear that innovative regulatory policies are required to expand the funding models for repurposing.

## Conclusions

Drug repurposing remains an important drug development strategy in discovering the untapped potential of existing drugs to enhance RT. Only two drugs have been successfully repurposed as radiosensitisers, nimorazole and nicotinamide, of which use is still largely limited to Denmark and the UK, respectively. From the 125 trials that investigated non-cancer drugs as radiosensitisers, we identified multiple barriers to success, including poor trial reporting, absence of biomarkers and patient selection, suboptimal pharmacological properties, inappropriate trial design, lack or inadequate consideration of evidence, and limited funding. Although the outlook for repurposing drugs for RT may appear challenging due to the numerous obstacles highlighted, we remain cautiously optimistic about future success. The chances of future successes would be improved by generating and thoughtfully considering relevant clinical and preclinical data, the discovery of robust and practical methods of patient stratification, more careful trial design and changes in regulatory policies to improve trial reporting and increase financial incentives.

## Data Availability

All data relevant to the study are included in the article or uploaded as online supplemental information.
