# Supplementary material for: Repurposing radiosensitising medicines for radiotherapy: an overview
Source: BMJ Oncol. 2024 Jan 17;3(1):e000192. doi: 10.1136/bmjonc-2023-000192 (PMC11235008; doi:10.1136/bmjonc-2023-000192)
Supplement: Supplementary data [file bmjonc-2023-000192supp001.pdf]

Supplementary Table 1. List of non-cancer drugs investigated in clinical trials for their radiosensitising properties.

Drugs	Main indication(s)	Phase(s)	Trial Identifier	Cancer type(s)	Trial description	Patient numbers	Status/ Results (as per August 2023)
Acetazolamide	Diuretic; anticonvulsant	1	NCT03467360	Small Cell Lung Cancer	Phase I study with a non-randomized, open-label extension cohort, evaluating the safety of acetazolamide in combination with platinum and etoposide-based radiochemotherapy for localized small cell lung cancer	Target: 30	Recruiting. Estimated study completion year: 2024 (Last updated 20 January 2023 on ClinicalTrials.gov)
Atorvastatin	Lipid-lowering drug	2	NCT02029573	Glioblastoma	Single-arm phase II trial on the safety and efficacy of atorvastatin in combination with RT and temozolomide in glioblastoma	Enrolled: 36	Completed. Results: Improvement in progression-free survival at 6 months (PFS-6) (primary endpoint) not statistically significant. (DOI: 10.1007/s10637-020-00992-5)
Atovaquone	Antimalarial	1	ARCADIAN; NCT04648033	NSCLC	Phase I trial to determine the maximum tolerated dose (MTD) of atovaquone atovaquone in combination with concurrent chemoradiotherapy (CRT) in locally advanced NSCLC	Target: 21	Recruitment completed. Estimated study completion year: 2024. (Last updated 11 May 2023 on ClinicalTrials.gov)
Celecoxib	Anti-inflammatory	1	NCT00177853	Pancreatic cancer	Single arm phase I study to investigate safety and efficacy of celecoxib with irinotecan and concurrent RT in preoperative pancreatic cancer	Target: 23	Terminated (Unknown cause)
			-	Nasopharyngeal	Phase I trial investigating the safety of concurrent celecoxib and RT in locoregionally advanced nasopharyngeal carcinoma	Enrolled: 34	Completed. Results: Well tolerated. (DOI: 10.1016/j.oraloncology.2011.06.002)
			-	Unresectable NSCLC	Phase I clinical trial of thoracic RT and concurrent celecoxib for inoperable/unresectable non-small cell lung cancer	Enrolled: 47	Completed. Results: Well tolerated at FDA-approved dose. (DOI: 10.1158/1078-0432.CCR-04-1741)
		1&2	-	Oesophageal cancer	Phase 1/II trial assessing the maximum tolerated dose (MTD) of celexocib combined with CRT (cisplatin, irinotecan, and RT) in locally advanced oesophageal cancer	Enrolled: 13 (before termination)	Terminated (External safety concerns - e.g. cardiovascular toxicities and thromboembolic risks - from the Food and Drug Administration (FDA) and Therapeutic Goods Administration (Australia)) Results: Primary endpoint (MTD) not reached. (DOI: 10.1007/s10637-006-9016-5)
		2	NCT00181532	NSCLC	Randomised, double-blinded placebo-controlled phase II trial to evaluate tumour response and toxicity profile in celecoxib and concurrent RT in stage II-III NSCLC	Enrolled: 40 (target of 102)	Terminated (Slow accrual) Results: Formal statistical analysis not performed. Hazard ratio is 0.62 in favour of celecoxib arm for OS. (DOI:https://doi.org/10.1016/j.radonc.2007.05.008)
			NCT00520091	Oesophageal Cancer	Non-randomised two-arm phase II trial of irinotecan, cisplatin, and RT with or without celecoxib in stage II to IV oesophageal cancer	Target: 14	Completed. Results unknown. (Last updated 18 May 2012 on ClinicalTrial.gov)
			NCT00137852	Oesophageal Cancer	Non-randomised, single-arm phase 2 trial studying the response rate and safety of adding celecoxib to neoadjuvant cisplatin-irinotecan chemoradiation for	Enrolled: 40	Completed. Result: Whilst the addition of celecoxib was tolerable, OS was comparable to using neoadjuvant cisplatin-irinotecan chemoradiation alone in historical studies (DOI: 10.1186/s12885-016-2485-9)

					operable oesophageal cancer		
			JPRN-UMIN000012818	Locally advanced rectal cancer	Non-randomised, single-arm, single center phase I/II trial studying MTD and efficacy of preoperative chemoradiotherapy using S-1 combined with celecoxib for advanced lower rectal cancer	Enrolled: 21	Completed. Results: Primary endpoint of 30% target pathological complete remission (pCR) rate was not met (15.8%), indicating no synergistic or additive effects. (DOI: 10.23922/jarc.2018-026)
			NCT00068770	Glioblastoma	Non-randomised, two-arm phase II trial studying the effects of hepatic enzyme-inducing antiseizure drugs (+EIASD) on the pharmacokinetics of celecoxib in patients with newly diagnosed GBM receiving RT. Those received EIASD (+EIASD) were compared against those who did not (-EIASD).	Enrolled: 35	Terminated after the EORTC trial (DOI: 10.1056/NEJMoa043330) showed temozolomide (TMZ) and RT conferred significant survival in this population). Results: Well tolerated. No differences between pharmcokinetic parameter between the groups (DOI: 10.1215/15228517-2007-055)
			NCT01503385	Unresectable NSCLC	Single-centre, open-label, randomised phase II trial of cisplatin/etoposide and concurrent RT wth or without celecoxib in patients with unresectable locally advanced NSCLC	Enrolled: 100	Completed. Results: No improvement in OS (primary endpoint). (DOI: 10.1001/jamanetworkopen.2019.18070)
			-	NSCLC	Phase II study of celecoxib in combination with paclitaxel, carboplatin, and radiotherapy for patients with inoperable stage IIIA/B non-small cell lung cancer	Enrolled: 17	Terminated because predetermined goal of 80% overall response rate was not met. Results: No improvement in survival. (DOI: 10.1158/1078-0432.CCR-08-0629)
			-	Pancreatic cancer	Single-arm phase II trial investigating toxicity and efficacy of short intensive uracil/tegafur based CRT combined with celecoxib in locally advanced pancreatic cancer	Enrolled: 83.	Completed. Results: Poorly tolerated; no partial or complete response observed; poor survival consistent with other studies with 5-fluouracil and CRT (DOI: 10.1016/j.radonc.2010.10.016)
			-	Rectal cancer	Single-arm phase II trial investigating the effects of celecoxib combined with preoperative chemoradiation (uracil-tegafur and RT) for locally advanced rectal cancer	Enrolled: 35	Completed. Results: Complete pathological remission (primary endpoint) target of >15% was met. High incidence (49%) of rash that led to non-compliance. (DOI: 10.1007/s00384-007-0407-7)
Chloroquine	Antimalarial	1	NCT01727531	Brain metastases	Single-arm study to evaluate efficacy of adding chloroquine to whole brain RT in patients with brain metastases and whether the IDO2 genetic status informs efficacy of his combination	Enrolled: 20	Completed. Results (abstract summary only, no peer-reviewed publication available): Inconclusive (DOI:https://doi.org/10.1016/j.ijrobp.2011.06.1313)
			NCT02378532; CHLOROBRAIN	Glioblastoma	Single-centre, open label, dose-finding phase I trial for the addition of chloroquine to temozolomide and concurrent RT in glioblastoma	Enrolled: 13	Completed. Results: Well tolerated, MTD established. (DOI: 10.1080/15548627.2020.1816343)
			NCT04397679	Glioblastoma	Open-label phase I trial for partial brain RT, temozolomide, chloroquine, and tumour treating field therapy for newly diagnosed glioblastoma	Target: 10	Recruiting. Estimated study completion year: 2025 (Last updated 24 April 2023 on ClinicalTrials.gov)
			NCT00969306; Chloroquine IV	SCLC	Non-randomised phase I trial studying toxicity and response of adding chloroquine to cisplatin-etoposide in extensive disease SCLC or to concurrent	Enrolled: 5	Terminated (Slow accrual)

					RT and cisplatin-etoposide in limited disease SCLC		
		2	NCT01894633	Brain Metastases	Randomized, double-blind, placebo-controlled phase II study of whole-brain irradiation with concomitant chloroquine for brain metastases	Enrolled: 73	Completed. Results: Chloroquine used concurrently with RT is well tolerated and improves local control and PFS compared to the control arm (DOI: 10.1186/1748-717X-8-209)
			NCT02432417; CHLOROBRAINII	Glioblastoma	Randomised controlled phase II trial for the addition of chloroquine to CRT for glioblastoma with endpoint of PFS-6.	Target: 156	Estimated study completion year: 2025 (Last updated 12 April 2022 on ClinicalTrials.gov)
		3	NCT00224978	GBM	Randomized, placebo-controlled, double-blind phase III trial for the addition of chloroquine as adjuvant to conventional treatment for GBM	Enrolled: 30	Completed. Results: Improvement in median survival (primary endpoint) did not reach statistical significance (DOI: 10.7326/0003-4819-144-5-200603070-00008)
Cidofovir	Antiviral	1	NCT00811408	Cervical	Phase I trial evaluating cidofovir in combination with CRT for stages IB2 to IVA cervical cancer	Enrolled: 15	Terminated (likely due to slow accrual) (DOI: 10.18632/oncotarget.8224)
Darbepoetin alfa	Treatment for anaemia	3	–	HNSCC	Randomised controlled phase III trial comparing HNSCC patients undergoing accelerated RT with or without darbepoetin alfa	Enrolled: 522 (target of 600)	Terminated after interim analysis showed inferiority of treatment arm. Results. Darbepoetin alfa-treated patients had worse loco-regional control (DOI: https://doi.org/10.1016/j.radonc.2018.02.018
Dimethyl fumarate (DMF)	Treatment for psoriasis, multiple sclerosis	1	NCT02337426	GBM	Phase I trial to determine RP2D for dimethyl fumarate in combination with TMZ and concurrent RT for newly diagnosed GBM.	Enrolled: 12	Completed. Results: Well tolerated, RP2D established (DOI: 10.1093/noajnl/vdz052)
Disulfiram	Alcohol antagonist drug	1	NCT02715609	Glioblastoma	Phase I/II dose-escalation and dose-expansion study of disulfiram/copper with concurrent RT and TMZ in newly diagnosed glioblastoma	Enrolled: 18	Ongoing. Estimated study completion year: 2024
Enoxaparin	Anticoagulant	2	NCT03254511; IRCT2016070628814N1	Oesophageal cancer	Randomised, single-blind phase II trial investigating the addition of enoxaparin to neoadjuvant CRT (paclitaxel-cisplatin and RT) for oesophageal cancer	Target: 100	Status unknown. (Last updated 22 August 2017 on ClinicalTrials.gov)
Erythropoietin	Treatment	3	RTOG 99-03	HNSCC	Randomised controlled phase III trial by the	Enrolled: 148	Terminated (interim analysis showed futility). Results: Non-statistically

(EPO)	for anaemia secondary to chronic kidney disease				Radiation Therapy Oncology Group (RTOG) investigating the outcomes of anaemic patients with HNSCC receiving RT with or without erythropoietin (epoetin α)	(Target: 372)	significant trend towards worse loco-regional failure rate in EPO arm. (DOI: 10.1016/j.ijrobp.2007.04.063)
			-	HNSCC	Randomised, double-blind, placebo-controlled phase III trial on the addition of EPO to treat HNSCC patients with anaemia undergoing RT	Enrolled: 351	Completed. Results: Epoetin β is associated with statistically significant worse locoregional PFS (primary endpoint) compared to placebo. (DOI:https://doi.org/10.1016/S0140-6736(03)14567-9)
			NCT00348738	Cervical cancer	Randomised controlled phase III trial on disease specific survival, tumour response, and local control in patients with cervical cancer who receive definitive RT with or without EPO	Target: 300	Status unknown. (Last updated 2 November 2007 on ClinicalTrials.gov)
			NCT00017004	Cervical cancer	Randomised phase III trial studying the efficacy of maintaining haemoglobin level above 120 g/l with EPO versus above 100 g/l without EPO in anaemic patients receiving concurrent RT and cisplatin for cervical cancer	Enrolled: 114 (< 25% of target of 460)	Terminated (Potential concern by study sponsor regarding thromboembolic event with study drug) Results: Inconclusive (DOI: 10.1016/j.ygyno.2007.10.011)
			-	HNSCC	Randomised open label phase III trial evaluating effects of epoetin alfa on local disease-free survival in patients receiving RT with curative intent	Enrolled: 301	Completed. Results: No benefit in survival with the addition of epoetin alfa to RT compared to RT alone. (DOI: 10.1200/JCO.2009.22.3693)
Hydralazine	Antihypertensive	2	NCT00404326	Cervical cancer	Single-arm phase II trial studying safety and efficacy of hydralazine in combination with valproate and cisplatin CRT in cervical cancer	Enrolled: 22	Completed. Results (abstract summary only, no peer-reviewed publication available): Well tolerated (DOI: https://doi.org/10.1186/1471-2407-7-S1-A28)
Hydroxychloroquine (HCQ)	Antimalarial; treatment for rheumatoid arthritis and SLE	1	NCT01417403	Solid tumours with bone metastases	Phase I trial to establish MTD of HCQ in patients with solid tumours undergoing RT for bone metastases	Enrolled: 10	Terminated (published data established safety of using higher dose than dose in current study – see DOI: 10.4161/auto.28984)
		1&2	NCT00486603	GBM	Dose-escalation phase I and non-comparative phase II trial to assess safety and efficacy of HCQ in conjunction with RT and concurrent and adjuvant TMZ in patients with newly diagnosed GBM.	Enrolled: 16 in phase I, 76 in phase II	Completed. Results: No significant improvement in OS when compared to cohort from EORTC trial (TMZ + RT vs RT alone) (DOI: 10.4161/auto.28984)
		2	NCT01602588	High grade gliomas (HGG)	Randomised, two-arm phase II trial investigating survival at one year with the addition of HCQ to short course RT (SCRT) in patients aged ≥70 years with HGG	Enrolled: 54	Terminated (as advised by the IDMC due to differences in survival) Results: Worse survival in HCQ arm compared to control arm. (DOI: 10.1093/noajnl/vdaa046)
			NCT04011410	Oligometastatic prostate cancer	Single-arm, non-blinded phase II trial to evaluate tumour suppressor PAR-4 levels from baseline in patients with oligometastatic prostate cancer treated with three months of HCQ in combination with RT or surgery	Target: 20	Ongoing. Estimated study completion year: 2026
			NCT01494155	Resectable pancreatic cancer	Single-arm phase II trial studying the efficacy of SCRT with proton or photon beam capecitabine and HCQ for resectable pancreatic cancer	Target: 50	Status unknown. Estimated study completion: January 2023. (Last updated 2 February 2021 on ClinicalTrials.gov)

Indinavir	Antiretroviral	2	NCT00637637	Brain metastases	Randomised, two-arm phase II trial on the addition of indinavir and ritonavir to external beam RT for patients with brain metastases	Target: 60	Status unknown. (Last updated 12 August 2013 on ClinicalTrials.gov)
Losartan	Antihyperten sive	1	NCT04106856; SHAPER	Unresectable Pancreatic Cancer	Phase I trial of the addition of losartan to hypofractionated RT after chemotherapy in patients with borderline resectable or locally advanced unresectable pancreatic cancer	Target: 20	Recruiting. Estimated study completion year: 2026
		2	NCT05861336; OVERPASS	Pancreatic Cancer	Single-arm phase II trial of GEM+Nab-Paclitaxel and losartan followed by stereotactic RT for locally advanced pancreatic cancer	Target: 34	Not yet recruiting. Estimated study completion year: 2029
Lovastatin	Lipid-lowering drug	1&2	-	Anaplastic astrocytoma and GBM	Phase I-II trial of lovastatin with or without RT in patients with for anaplastic astrocytoma and GBM	Enrolled: 18	Completed. Results: Well tolerated (DOI: 10.1097/00000421-199812000-00010)
Mebendazole	Anthelmintic	1	NCT01837862	Paediatric glioma	Phase I trial of the addition of mebendazole to chemotherapy combination (carboplatin, vincristine, temozolomide) in patients with low-grade glioma or to CRT (bevacizumab, irinotecan, and RT) in patients with high-grade glioma/pontine glioma	Target: 36	Recruiting. Estimated study completion year: 2025
			-	HGG	Reverse swing-M, phase 1 study of repurposing mebendazole in recurrent HGG	Enrolled: 11	Completed. RP2D established (DOI: 10.1002/cam4.3094)
Melatonin	Sleeping drug	2	NCT00031967; RTOG 0119	Brain metastases	Randomized, two-arm phase II trial of melatonin and RT in patients with brain metastases (Arm I: melatonin taken in the morning versus Amr II: taken in the evening)	Enrolled: 130	Completed. Results: Both treatment arms had worse median survival (3.4 and 2.8 months, in morning and evening melatonin treatment arms respectively), compared to RTOG historical control survival of patients treated for brain metastases (4.1 months). (DOI: 10.1016/j.ijrobp.2007.01.012)
Metformin	Hypoglycaem ic	1	NCT02149459	Brain tumour	Phase I trial studying toxicity profile and compliance of metformin, moderately low carbohydrate diet, and RT	Target: 18	Status unknown. (Last updated 27 October 2017 on ClinicalTrials.gov)
			NCT02153450	Pancreatic cancer	Open label pilot, single-centre, non-randomized phase I trial to assess tolerability and preliminary activity of the combination of stereotactic body radiation therapy (SBRT) with metformin for resectable and locally-advanced pancreatic/periampullary cancers	Enrolled: 8	Completed. Results unknown. (Last updated 26 June 2020 on ClinicalTrials.gov)
		1&2	NCT02949700	HNSCC	Single-arm phase I/II trial of metformin in combination with cisplatin and RT in HNSCC	Enrolled: 26	Completed. Results unknown. (Last updated 29 June 2023 on ClinicalTrials.gov)
			NCT04536805; REPAIRGETUGP 16	Prostate cancer	Randomised, three-arm trial to study safety and efficacy of adding metformin to stereotactic ablative reirradiation to patients with relapse in previously	Target: 46	Recruiting. Estimated study completion year: 2028

					irradiated prostate bed		
		2	NCT02945813; SAKK 08/15 - PROMET	Prostate cancer	Randomized, two-arm phase II trial of salvage RT with or without metformin in non-diabetic patients with prostate cancer after prostatectomy	Enrolled: 111 (Target: 170)	Terminated early by the SAKK Board due to financial reasons. Results (abstract summary only, no peer-reviewed publication available): No significant improvement in time to progression (TTP) in arm treated with metformin compared to control arm. Trial was underpowered due to premature termination (DOI: 10.1200/JCO.2023.41.6_suppl.353)
			NCT04275713; METOXY-LACC	Cervical cancer	Randomized, two-arm phase II trial studying altered tumour oxygenation in patients with LACC receiving cisplatin and RT with or without metformin Tumour oxygenation will be evaluated by gene signatures and MRI- parameters.	Target: 90	Recruiting. Estimated study completion year: 2025.
			NCT02394652	Cervical cancer	Randomized, two-arm phase II trial evaluating tumour oxygenation in patients with LACC receiving radical RT and concurrent cisplatin chemotherapy with or without metformin.	Enrolled: 20	Completed. Results: Reduction in tumour hypoxia as evaluated on FAZA-PET scan (DOI: 10.1158/1078-0432.CCR-22-1665)
			NCT04170959; RADFORMIN	NSCLC	Observational lead-in phase I and a randomized, three-arm phase II trial studying the addition of metformin to definitive RT in patients with inoperable stage III NSCLC	Enrolled: 3	Terminated (Loss of external funding)
			NCT02285855	NSCLC	Randomised, two-arm, placebo-controlled phase II trial evaluating the addition of metformin to SBRT in NSCLC	Enrolled: 27 (Target: 70)	Terminated (Slow accrual)
			NCT02473094; NEOMETRE	Rectal cancer	Randomized, two-arm, placebo-controlled phase II trial to evaluate the efficacy and tolerability of metformin in addition to CRT for the preoperative locally advanced rectal carcinomas.	Enrolled: 3 (Target: 98)	Terminated (Slow accrual)
			NCT02186847; NRG-LU001	NSCLC	Randomised, two-arm phase II trial investigating outcomes in patients with stage III NSCLC who received either CRT (carboplatin-paclitaxel and RT) alone or CRT with metformin	Enrolled: 170	Completed. Results: Worse PFS at 1 year (primary endpoint) in treatment arm. (DOI: 10.1001/jamaoncol.2021.2318)
			NCT02115464; OCOG-ALMERA	NSCLC	Randomised, open-label, two-arm phase II trial evaluating outcomes in NSCLC patients receiving either CRT (cisplatin-based regimen) alone or CRT and metformin	Enrolled: 54 (Target: 96)	Terminated (Slow accrual) Results: Addition of metformin to CRT was associated with worse outcomes (PFS at 1 year and OS) and increased toxicities compared to CRT alone. (DOI: 10.1001/jamaoncol.2021.2328)
Metoclopramid e	Antiemetic	1&2	-	Lung SCC	Phase I/II evaluation of metoclopramide in patients with inoperable lung SCC receiving RT. The two arms studied were RT and metformin three times a week versus RT and metformin five times a week.	Enrolled: 23	Completed. Results: Well tolerated; tumour response and survival positively correlated with total and weekly metoclopramide received. (DOI: 10.1016/0959-8049(95)00424-6)
Metronidazole	Antibiotic	2&3	NCT01937650	Cervical cancer	Randomised, two-arm, placebo-controlled trial on tumour volume reduction in cervical cancer patients receiving RT and metronidazole versus RT alone	Target: 38	Completed. Results unknown. (Last updated 9 September 2013 on ClinicalTrials.gov)

Mycophenolate mofetil (MMF)	Immunosuppressant	0&1	NCT04477200	Glioblastoma	Phase 0/I trial of MMF combined with RT to overcome glioblastoma treatment resistance by targeting de-novo purine metabolism.	Target: 68	Ongoing. Estimated study completion year: 2027.
Nelfinavir	Antiretroviral	1	NCT01068327	Pancreatic cancer	Phase I trial of stereotactic RT, nelfinavir mesylate, gemcitabine hydrochloride, leucovorin calcium, and fluorouracil in patients with locally advanced pancreatic cancer	Enrolled: 46	Completed. Results: Well tolerated, MTD established. (DOI: 10.1016/j.radonc.2018.11.002)
			NCT02363829	LACC	Phase I trial of nelfinavir added to cisplatin concurrent with pelvic RT for LACC (II-IVA)	Enrolled: 13	Completed. Results: Well tolerated, RP2D established. (DOI: 10.1002/cncr.33449)
			NCT04169763	Vulvar cancer	Phase I trial of nelfinavir, cisplatin, and external beam RT for unresectable locally advanced vulvar cancer	Target: 18	Estimated study completion: Dec 2023. (Last updated 21 September 2020 on ClincialTrials.gov)
			NCT00915694	Glioma	Phase I trial with nelfinavir mesylate, RT, and TMZ in patients with WHO grade IV glioma	Enrolled: 15	Terminated (Slow accrual)
			NCT01020292	Glioma	Phase I trial of nelfinavir and concurrent RT and TMZ in patients with WHO grade IV glioma	Enrolled: 21	Completed. Results: Well tolerated (DOI: 10.1007/s11060-013-1303-3)
			ARC I	Pancreatic cancer	Phase I trial combining nelfinavir with gemcitabine of different doses with cisplatin-based CRT in patients with pancreatic cancer	Enrolled: 12	Completed. Results: Well tolerated. (DOI: 10.1200/JCO.2007.15.2355)
		1&2	NCT01447589	NSCLC	Phase I dose-escalation study of nelfinavir, given concomitantly with radical RT for NSCLC	Enrolled: 0	Withdrawn due to failure to recruit
			NCT01086332	Pancreatic cancer	Phase I/II trial of nelfinavir and concurrent RT and gemcitabine for locally advanced pancreatic cancer	Enrolled: 7	Terminated (Toxicities and lack of funding)
			NCT00589056	NSCLC	Open-label, single-group phase I/II trial on the addition of nelfinavir to CRT (cisplatin,etoposide, and RT) in inoperable stage III NSCLC	Enrolled: 38	Completed. Results: Well tolerated. (DOI: 10.1001/jamaoncol.2019.2095)
			NCT02024009; SCALOP-2	Pancreatic cancer	Phase I/II trial of pancreatic cancer patients receiving gemcitabine and nab-paclitaxel randomised into one of five arms - further chemotherapy, 50.4 Gy capecitabine-based CRT with or without nelfinavir or 60Gy capecitabine-based CRT with or without nelfinavir	Enrolled: 186 (27 in phase 1; 159 in phase 2) (Target: 289)	Terminated (Closure of nelfinavir arm advised by ISDMC due to futility. Results (abstract summary only, no peer-reviewed publication available): Addition of nelfinavir did not improve survival. (PMID: WOS:000806759200078)
			NCT01108666	NSCLC	Phase I/II trial of proton beam radiation with concurrent chemotherapy and nelfinavir for inoperable stage III NSCLC	Enrolled: 7 (Target: 72)	Terminated (Slow accrual)
		2	NCT00694837	Glioblastoma	Phase I trial to determine the MTD of nelfinavir as adjuvant with CRT for glioblastoma	Enrolled: 6	Completed. Results unknown. (Last updated 10 April 2015 on ClinicalTrials.gov)
			NCT02207439	HNSCC	Single-arm, two-period phase II Trial of nelfinavir with concurrent CRT in HNSCC	Enrolled: 17	Completed. Results awaited.

			EUCTR2010-020621-40-GB; SONATINA	Rectal cancer	Non-randomised single-arm phase II trial evaluating the safety of adding nelfinavir to RT in neoadjuvant therapy for rectal cancer	Enrolled: 10	Completed. Results: Well tolerated (DOI: 10.1158/1078-0432.CCR-15-1489)
			EudraCT 2008-006302-42; ARC-II	Pancreatic cancer	Single-arm phase II trial of nelfinavir in combination with CRT for locally advanced inoperable pancreatic cancer	Enrolled: 23	Terminated (Non-availability of nelfinavir in Europe) Results: Well tolerated, OS better than historical survival rates (DOI: 10.1016/j.radonc.2016.03.021)
		3	NCT03256916; NELCER	Cervical cancer	Randomised, two-arm, open-label phase III trial on the addition of nelfinavir to concurrent cisplatin, pelvic external beam RT and brachytherapy in patients with LACC	Target: 348	Recruiting. Estimated study completion year: 2025.
Nicotinamide	Treatment for niacin deficiency	2	-	Bladder cancer	Three-arm phase II trial involving patients with bladder cancer who would be included in one of three arms – carbogen, nicotinamide, or both carbogen and nicotinamide – whilst receiving radical RT	Enrolled: 30	Completed. Results: Well tolerated in all three groups. Favourable rates of local control at 6 momths. (DOI: 10.1038/bjc.1997.372)
			-	GBM	Two-arm phase II trial evaluating the toxicity and efficacy of RT and chemotherapy (carmustine) with or without carbogen and nicotinamide in inoperable biopsy-proven GBM	Enrolled: 33	Completed. Results: Carbogen and nicotinamide only tolerated in half of patients in treatment arm. No significant differences in OS between treatment groups (DOI: 10.1016/s0167-8140(03)00007-0)
			ISRCTN08912168; PROCON	Prostate cancer	Single-arm phase II trial of RT in conjunction with carbogen and nicotinamide (CON) in prostate cancer	Enrolled: 50	Completed. Results (abstract summary only, no peer-reviewed publication available): Well tolerated (https://christie.openrepository.com/handle/10541/624399)
		3	NCT00033436	Bladder cancer	Randomized phase III trial to compare the effectiveness of RT with or without carbogen and niacinamide (CON) in patients with locally advanced bladder cancer.	Enrolled: 333	Completed. Results: CON + RT showed nonsignificant improvement in cystoscopic control at 6 months compared to RT alone (DOI: 10.1200/JCO.2010.28.4950)
			NCT00147732	Laryngeal cancer	Randomised phase III clinical trial comparing accelerated radiotherapy (AR) with accelerated radiotherapy plus carbogen and nicotinamide (ARCON) in clinical stage T2-4 laryngeal carcinoma.	Enrolled: 345	Completed. Results: ARCON showed no significant improvement in local tumour control (primary endpoint) compared to AR. (DOI: 10.1200/JCO.2011.35.9315
Nimorazole	Antifungal	1	-	HNSCC	Phase I study of nimorazole in patients with HNSCC	Enrolled: 17	Completed. Results: Well tolerated (DOI: 10.1016/0360-3016(84)90545-5)
		2	DAHANCA 18	HNSCC	Single-arm phase II trial on locally advanced head and neck cancer treated with accelerated radiotherapy, nimorazole and weekly cisplatin	Enrolled: 227	Completed. Results: Well tolerated. Better loco-regional control (primary endpoint) of 80% compared to historical DAHANCA data (70%) (DOI: 10.3109/0284186X.2014.992547)
			DAHANCA 28	HNSCC	Phase I/II feasibility study of hyperfractionated, accelerated radiotherapy with concomitant cisplatin and nimorazole (HART-CN) for patients with locally advanced, HPV/p16-negative squamous cell	Enrolled: 50	Completed. Results: Combined treatment was feasible but was associated with significant acute toxicities. (DOI: 10.1016/j.radonc.2020.03.025)

					carcinoma of the oropharynx, hypopharynx, larynx and oral cavity		
			-	HNSCC	Single-arm phase II trial to determine efficacy of hyperfractionated accelerated radiation therapy (CHART)/nimorazole regimen.	Enrolled: 61	Completed. Results: Local control rates in CHART + nimorazole were better than those previously seen with CHART alone (DOI: 10.1016/s0167-8140(02)00284-0)
			CTRI/2019/02/017477	Cervical cancer	Randomised, two-arm phase II trial of nimorazole with CRT for LACC compared to CRT alone	Target: 196	Status unknown. (Last updated 22 November 2019 on trial register CTRI)
		3	DAHANCA protocol 5-85	HNSCC	Randomized, placebo-controlled, double-blind phase III study by the Danish Head and Neck Cancer Study (DAHANCA) on nimorazole with RT in supraglottic larynx and pharynx carcinoma	Enrolled: 422	Completed. Results: Statistically significant improvement in locoregional control (primary endpoint) in those who received nimorazole compared to placebo. (DOI: 10.1016/s0167-8140(97)00220-x)
			NCT01950689; NIMRAD	HNSCC	Randomised, placebo-controlled trial of nimorazole and RT versus RT alone in patients with locally advanced HNSCC unsuitable for synchronous chemotherapy or cetuximab	Enrolled: 338	Completed. Results (abstract summary only, no peer-reviewed publication available): Addition of nimorazole did not improve loco-regional control or survival compared to placebo (DOI: 10.1200/JCO.2023.41.16_suppl.6006)
			NCT01507467; IAEA-HypoX	HNSCC	Randomised, two-arm phase III trial of accelerated RT with or without nimorazole in HNSCC	Enrolled: 104 (Target: 600)	Terminated (Slow accrual) Results: Inconclusive as underpowered; improvements in loco-regional tumour control and OS with the addition of nimorazole did not reach statistical significance. (DOI: 10.1016/j.radonc.2015.04.005)
			NCT01880359; EORTC-1219	HNSCC	Randomized, placebo-controlled phase III trial of accelerated fractionated CRT with or without nimorazole, using a 15-gene signature for hypoxia in the treatment of HNSCC	Target: 640	Status unknown. Estimated completion time: January 2023 (Last updated 10 May 2022 on ClinicalTrials.gov)
			DAHANCA30; NCT02661152	HNSCC	Randomized non-inferiority phase III trial of hypoxia-profile guided hypoxic modification of RT with nimorazole in patients with HNSCC	Target: 1252	Ongoing. Estimated study completion: December 2023.
Nitroglycerin	Vasodilator	1	NCT01407107	Rectal cancer	Phase I dose-escalation trial of nitroglycerin in addition to 5-fluorouracil and RT for neo-adjuvant treatment of operable rectal cancer	Enrolled: 13	Completed. Results: Well tolerated (DOI: 10.1016/j.surg.2015.04.007)
		2	NCT04338867	Brain metastases in NSCLC	Randomised controlled, open-label phase II trial evaluating the addition of nitroglycerin to whole intracranial RT for brain metastases in NSCLC	Enrolled: 96	Completed. Results: Treatment arm had better intracranial objective response rate (iORR) and intracranial PFS compared to control arm. (DOI: 10.1016/j.ijrobp.2022.02.010)
			NCT00886405	NSCLC	Single-arm phase II study with concurrent CRT (vinorelbine + cisplatin) with nitroglycerin for locally advanced NSCLC	Enrolled: 35	Completed. Results: Well tolerated. OS was better (54%) than historical data (30%) from SWOG 8805 (DOI: 10.1016/j.radonc.2014.01.021)
			NCT01210378	NSCLC	Single-arm phase II trial evaluating 2-year overall survival (OS) (primary endpoint) in stage IB-IV NSCLC patients treated with radical (chemo-) RT and nitroglycerin patch during RT	Stopped at 42	Terminated (Accrual stopped following futility analysis – no reduction in tumour hypoxia on serial hypoxia PET/CT scans) (DOI: 10.1016/j.ctro.2019.12.002)
Papaverine	Smooth muscle relaxant - Vasodilator/ Anti-	1	NCT03824327	NSCLC	Papaverine and stereotactic body RT (SBRT) for NSCLC or lung metastases	Target: 24	Estimated study completion: December 2023.
			NCT05136846	NSCLC	Papaverine in combination with CRT for the treatment of stage II-III NSCLC	Target: 28	Estimated study completion year: 2024

	spasmodic						
Paricalcitol	Treatment for hyperparathyroidism	1	NCT01197664	Rectal cancer	Randomised, two arm phase I study of the addition of paricalcitol to standard CRT for resectable rectal cancers	Enrolled: 2	Terminated (Funding issue)
Pentoxifylline	Vasoactive agent	1	NCT00019058	High-grade astrocytoma or glioblastoma	Dose-escalation phase I study of hydroxyurea and pentoxifylline for treatment of glioblastoma with concurrent RT	Target: 34	Completed. Results unknown. (Last updated 29 April 2015 on ClinicalTrials.gov)
		(2)	-	NSCLC	Two-arm trial studying clinical outcomes of stage III NSCLC patients receiving RT with or without the concomitant use of pentoxifylline and alpha-tocopherol	Enrolled: 66	Completed. Results: Treatment arm had better OS rates at 1 and 2-years compared to control arm. (DOI: 10.1385/mo:23:2:185)
		3	-	NSCLC	Randomised, two-arm phase III trial evaluating radiation response and survival in NSCLC patients receiving RT and pentoxifylline versus RT alone.	Enrolled: 64	Completed. Results: Differences in radiation response and survival with the addition of pentoxifylline did not reach statistical significance (DOI: 10.1016/s0167-8140(00)00221-8)
Propranolol	Beta blocker	2	NCT04682158	Oesophageal	Randomised, three-arm phase II trial to evaluate the safety and efficacy of propranolol in combination with standard neoadjuvant/definitive CRT for oesophageal cancer	Target: 60	Recruiting. Estimated study completion year: 2026
Riluzole	Treatment for multiple sclerosis	1	NCT01018836	Brain metastases	Phase I trial of riluzole in combination with RT in patients undergoing whole brain RT for brain metastasis	Enrolled: 9	Terminated (Slow accrual)
Ritonavir	Antiretroviral	2	NCT00637637	Brain metastases	Randomised, two-arm phase II trial assessing efficacy in patients with brain metastases receiving RT with or without indinavir/ritonavir combination.	Target: 60	Status unknown. (Last updated 12 August 2013 on ClinicalTrials.gov)
Rosuvastatin	Lipid-lowering drug	2	NCT02569645; STARC	Rectal cancer	Single-arm phase II trial of rosuvastatin combined with standard CRT in the treatment of high-risk locally advanced rectal cancer	Enrolled: 45	Completed. Results (abstract summary only, no peer-reviewed publication available) (DOI: 10.1200/JCO.2023.41.4_suppl.131)
Simvastatin	Lipid-lowering drug	2	NCT02161822	Rectal cancer	Single-arm phase II study of neoadjuvant CRT with capecitabine plus simvastatin in patients with locally advanced rectal cancer	Enrolled: 61	Completed. Results: Efficacy has been assessed based on pCR responses compared to historical controls – no improvement seen. (DOI:10.4143/crt.2021.1527)
			NCT02104193	Brain metastases	Randomised controlled two-rm phase II trial studying the effect of simvastatin on the radiological response (primary endpoint) and clinical outcomes in patients with brain metastases treated with RT	Enrolled:50	Completed. Results: Addition of simvastatin did not improve radiological response, 1-year PFS, or 1-year OS rates. (DOI: 10.3727/096504016X14719078133528)

Sirolimus	Immunosuppressant	1	-	NSCLC	Phase I trial of sirolimus combined with RT and cisplatin in NSCLC	Enrolled: 7	Terminated (Loss of funding). Results: Well tolerated, MTD not established due to premature termination (DOI: 10.1097/JTO.0b013e3180cc2587)
Sulfasalazine	DMARD	1	NCT04205357	Glioblastoma	Dose escalation phase I trial combining sulfasalazine and gamma knife radiosurgery for recurrent glioblastoma	Target: 24	Completed. Results unknown. (Last updated 3 March 2023 on ClinicalTrials.gov)
Suramin	Anthelmintic	2	NCT00002639	Recurrent brain tumours	Phase II trial to study the effectiveness of suramin in treating patients with recurrent primary brain tumours following RT	Unknown	Completed. Results unknown. (Last updated June 21, 2013 on ClinicalTrials.gov)
			NCT00004073	GBM	Single-arm phase II study evaluating the safety and efficacy of suramin and concurrent RT in newly diagnosed GBM. OS was compared with historical data from the New Approaches to Brain Tumor Therapy (NABTT) GBM database	Enrolled: 55	Completed. Results: Well tolerated but no statistically significant improvements in OS compared to historical data. (DOI: 10.1215/S115285170300012)
Tolfenamic acid	Anti-inflammatory	1	NCT02159248	Pancreatic cancer	Dose-escalation phase I study of the addition of tolfenamic acid to gemcitabine and RT in patients with locally advanced or metastatic pancreatic cancer requiring definitive or palliative RT	Enrolled: 0 (Target: 24)	Withdrawn before enrolling any participants (cause unknown)
Tranilast	Antihistamine	2	NCT05626829	Nasopharyngeal carcinoma	Single-arm, open label phase II trial evaluating the addition of tranilast in the treatment of RT-resistant nasopharyngeal carcinoma	Target: 18	Recruiting. Estimated study end year: 2024
Valproic acid (VPA)	Anticonvulsant	1	NCT00437957	Brain metastases from solid tumours	Single-arm phase I trial of TMZ with VPA And whole brain RT for brain metastases from solid tumours in adults	Enrolled: 10	Terminated (Slow accrual)
			NCT01861990	Paediatric brain tumours	Single-arm, open label phase I trial for VPA in children with recurrent and progressive brain tumors	Enrolled: 0	Withdrawn before enrolment started (due to lack of feasibility)
		1&2	NCT01898104	Rectal cancer	Randomised, four-arm phase I/II trial of VPA and short-course RT plus capecitabine as preoperative treatment in low-moderate risk rectal cancer	Target: 86	Recruiting. Estimated study end year: 2024
			NCT01203735	NSCLC	Non-randomised phase I/II study to assess toxicity and efficacy of VPA in combination with concurrent CRT using vinorelbine and cisplatin for inoperable locally advanced NSCLC	Target: 20	Status unknown (Last updated 29 March 2011 on ClinicalTrials.gov)
		2	NCT00302159	Glioblastoma	Single-arm phase II trial evaluating the safety and efficacy of VPA in combination with TMZ and RT in patients with HGG. Primary endpoint of 1-year OS and 6-month PFS were compared against historical data from Stupp et al (DOI: 10.1056/NEJMoa043330)	Enrolled: 37	Completed. Results: VPA was well tolerated and had better OS and PFS compared to historical data. (DOI: 10.1016/j.ijrobp.2015.04.038)

			NCT01333631	Pancreatic cancer	Non-randomised, single-arm phase II trial evaluating VPA with concurrent gemcitabine CRT for unresectable locally advanced pancreatic cancer	Target: 20	Status unknown (Last updated 12 April 2011 on ClinicalTrials.gov)
			NCT00879437	Paediatric glioma	Single-arm phase II trial of VPA and RT, followed by maintenance VPA and bevacizumab in children with newly diagnosed HGG or brainstem gliomas. One-year EFS (primary endpoint) of 36% for GBM from the COGtrial and 17% for DIPG from Children's Cancer Group trial, CCG were used as as historical comparisons.	Enrolled: 38 (20 DIPG and 18 HGG)	Completed. Results: VPA was well tolerated but no improvement in EFS compared to historical data. (DOI: 10.1002/pbc.28283)
			NCT00404326	Cervical cancer	Phase II trial of hydralazine and VPA with concomitant cisplatin CRT for FIGO Stage III cervical cancer	Target: 18	Completed. Results unknown. (Last updated 28 November 2006 on ClinicalTrials.gov)
		3	NCT03243461; HIT-HGG-2013	HGG	Single-arm phase III trial of the HIT-HGG Study Group for the addition of valproic acid to CRT in children and adolescents < 18 years with HGG. Therapeutic efficiency and safety of VPA will be compared against data of the HIT-HGG-2007 trial (children and adolescents with same diseases, only treated with simultaneous TMZ CRT).	Target: 167	Recruiting. Estimated study completion: December 2023.
Warfarin	Anticoagulan t	3	-	SCLC	Randomized trial of chemotherapy and RT with or without warfarin in limited-stage small-cell lung cancer: a Cancer and Leukemia Group B study.	Enrolled: 369	Completed. Results: No statistically significant improvement in OS with the addition of warfarin to CRT. (DOI: 10.1200/JCO.1997.15.11.3378)
Zoledronic acid	Bisphosphon ate, treatment for osteoporosis	1&2	NCT03073785	Pancreatic cancer	Randomized, double-arm phase II study of the efficacy and safety of hypofractionated stereotactic RT and 5FU or capecitabine with and without zoledronic acid in patients With locally advanced pancreatic adenocarcinoma	Target: 44	Recruiting. Estimated study completion year: 2024
